# Revelation of candidate genes and molecular mechanism of reproductive seasonality in female rohu (*Labeo rohita* Ham.) by RNA sequencing

**DOI:** 10.1186/s12864-021-08001-6

**Published:** 2021-09-22

**Authors:** Sarika Jaiswal, Samiran Nandi, Mir Asif Iquebal, Rahul Singh Jasrotia, Sunita Patra, Gayatri Mishra, Uday Kumar Udit, Dinesh Kumar Sahu, U. B. Angadi, Prem Kumar Meher, Padmanav Routray, Jitendra Kumar Sundaray, Dhananjay Kumar Verma, Paramananda Das, Pallipuram Jayasankar, Anil Rai, Dinesh Kumar

**Affiliations:** 1 Centre for Agricultural Bioinformatics, ICAR-Indian Agricultural Statistics Research Institute, New Delhi, India; 2 ICAR- Central Institute of Freshwater Aquaculture, Bhubaneswar, Odhisa India

**Keywords:** *Labeo rohita*, Circannual, *Cyprinid*, Biological clock genes, Marker, Reproductive seasonality

## Abstract

**Background:**

Carp fish, rohu (*Labeo rohita* Ham.) is important freshwater aquaculture species of South-East Asia having seasonal reproductive rhythm. There is no holistic study at transcriptome level revealing key candidate genes involved in such circannual rhythm regulated by biological clock genes (BCGs). Seasonality manifestation has two contrasting phases of reproduction, i.e., post-spawning resting and initiation of gonadal activity appropriate for revealing the associated candidate genes. It can be deciphered by RNA sequencing of tissues involved in BPGL (Brain-Pituitary-Gonad-Liver) axis controlling seasonality. How far such BCGs of this fish are evolutionarily conserved across different phyla is unknown. Such study can be of further use to enhance fish productivity as seasonality restricts seed production beyond monsoon season.

**Result:**

A total of ~ 150 Gb of transcriptomic data of four tissues viz., BPGL were generated using Illumina TruSeq. *De-novo* assembled BPGL tissues revealed 75,554 differentially expressed transcripts, 115,534 SSRs, 65,584 SNPs, 514 pathways, 5379 transcription factors, 187 mature miRNA which regulates candidate genes represented by 1576 differentially expressed transcripts are available in the form of web-genomic resources. Findings were validated by qPCR. This is the first report in carp fish having 32 BCGs, found widely conserved in fish, amphibian, reptile, birds, prototheria, marsupials and placental mammals. This is due to universal mechanism of rhythmicity in response to environment and earth rotation having adaptive and reproductive significance.

**Conclusion:**

This study elucidates evolutionary conserved mechanism of photo-periodism sensing, neuroendocrine secretion, metabolism and yolk synthesis in liver, gonadal maturation, muscular growth with sensory and auditory perception in this fish. Study reveals fish as a good model for research on biological clock besides its relevance in reproductive efficiency enhancement.

**Supplementary Information:**

The online version contains supplementary material available at 10.1186/s12864-021-08001-6.

## Background

Reproductive rhythm of fish varies from prolific breeders to seasonal breeders. Though prolific breeder like common carp and zebra fish breed independent of season but vast majority of the freshwater fishes of Indian subcontinent breed seasonally during the monsoon season (June–August) when rainfall is heaviest [1]. This rhythm reflects evolution of differential biological clock.

In seasonal reproductive behaviour, BPG/ BPGL (Brain-Pituitary-Gonad-Liver) axis and fish biological clock is well known having entrainment of its biological clock with environment, temperature and food availability [2, 3]. Most active liver metabolism has been found in fish having potential to acquire bigger size thus liver transcriptome should be included in study of gonadal maturation by BPG (Brain-Pituitary-Gonad) axis [4]. Biological rhythm of seasonal breeding in fish is regulated by neuro-endocrine system, controlled by BPGL axis [5]. In order to decipher candidate genes regulating seasonal fish breeding, RNA sequencing of associated tissues are imperative. Such data can be used to construct gene regulatory networks (GRNs) to depict process of cell differentiation, metabolism, cell cycle and signal transduction in the biological system. Understanding dynamics of these networks can shed light on mechanism of reproductive seasonality.

Since circannual biological rhythm of seasonal reproduction is controlled by biological clock, regulating BPGL axis, thus RNA seq approach can also be used to discover biological clock genes. Biological clock genes originated 2.5 billion years ago and they are structurally conserved across phyla i.e., from lower vertebrates to higher vertebrates and can be used for their in silico identification. Differential expression of such genes across different tissues can be used to study control of reproductive seasonality and adaptation during species divergence [6]. Major carp of South-East Asia like *Labeo rohita* (Hamilton), which is an important freshwater aquaculture species having economic, ecological and cultural attributes would be a best model to study how seasonal manifestation of reproductive behavior is controlled by circannual biological clock.

Earlier attempts to investigate gene expression associated with seasonal breeding in carp fish, rohu were confined to limited genes (< 200) by microarray approach, limited ESTs (< 5000) comparing preparatory and post-spawning stages along with limited genomic resources in form of SSR (< 250) but without SNP [7]. In another such investigation by RNA Seq approach [8] limited reproductive genes were identified. This study enlists reproductive genes which are based on pooled RNA samples (of twelve tissues related to digestive and reproductive system) without differentially expressed genes (DEGs), thus lacks in identification of key candidate genes associated with reproductive seasonality. Even in one of the largest global consortium model fish transcriptome projects, FishT1K (https://www.fisht1k.org/) which is covering 124 species representing 46 orders, having six *cypriniformes* species but there is no data on carp fish, *Labeo rohita* which can be used as genomic resource to discover seasonality controlling genes along with biological clock genes (BCGs). RNA seq data of BPGL axis involved tissues can be used for evolutionary studies. A tissue-wise RNA sequencing comparison between two physiologically contrasting phases i.e.*,* post-spawning resting (PSR) and initiation of gonadal activity (IGA) can decipher the entire mechanism of reproductive seasonality. Such transcriptomic studies can reveal key candidate genes and their regulatory networks controlling neuroendocrine and physiological pathway operating in four different tissues, *namely,* BPGL.

The present work aims at revealing the candidate genes of seasonal biological rhythm of breeding and gene regulatory networks. It further aims to investigate differentially expressed biological clock genes (BCGs), along with their evolutionary conservation across jawless fish, cartilaginous fish (whale shark), bony fishes (zebrafish and European carp), amphibia, bird, reptile, monotreme (egg laying mammal), marsupials (young born) and placental mammals by comparative genomics. This study also aims to develop a genomic resource database of conserved BCGs of each phylum along with seasonality associated candidate genes in female carp fish, *Labeo rohita* (rohu).

## Results and discussion

### Reproductive phase identification by GSI estimation with ovarian morphology and histology

**Fig. 1:**
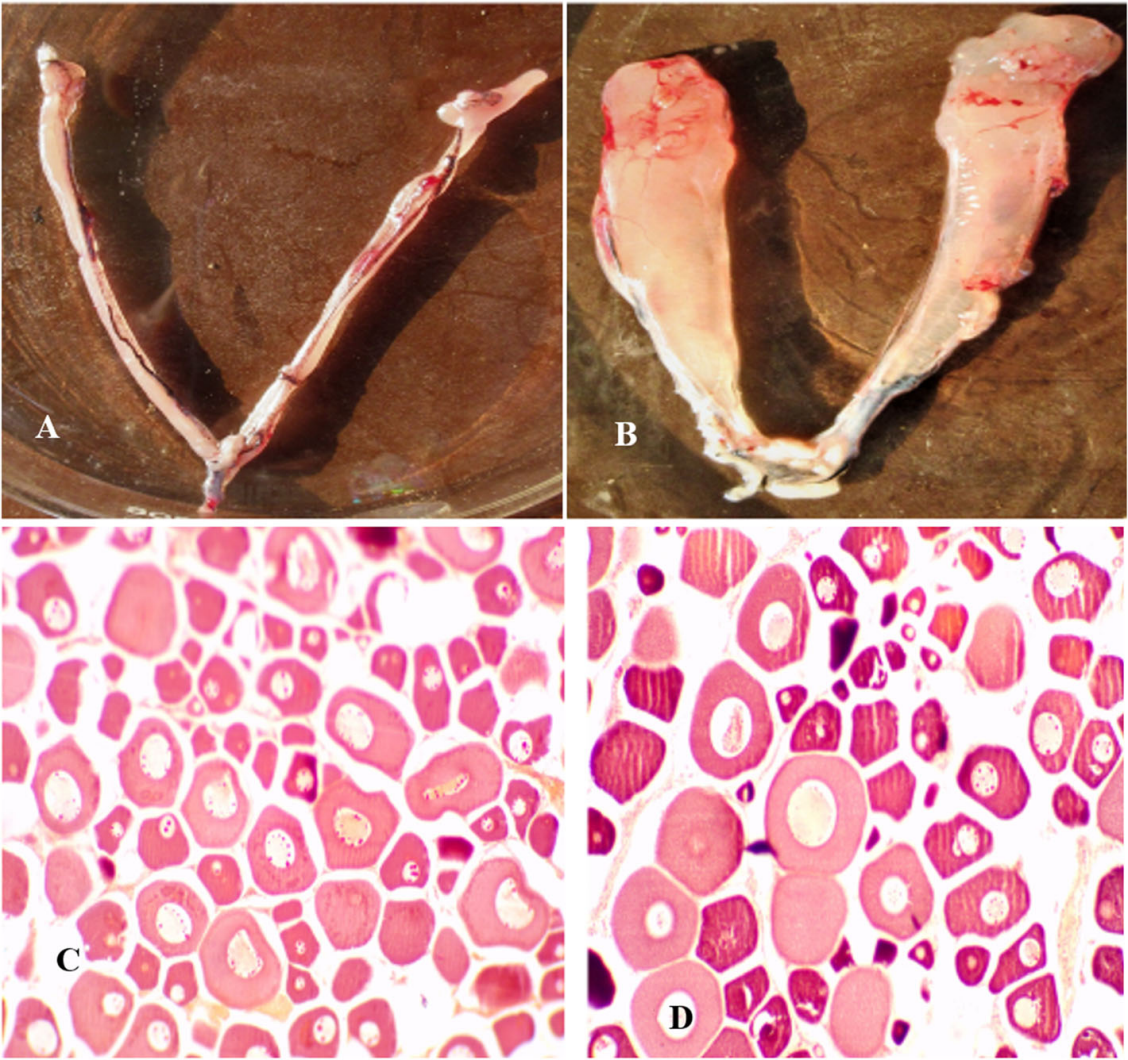
Morphological and histological view of rohu ovary in December and February (1A. Rohu ovary in December; 1B. Rohu ovary in February; 1C. Histological view of rohu ovary in December at 10X; 1D. Histological view of rohu ovary in February at 10X)

*Ovarian morphology*: The ovaries in December (Fig. 1A) appeared as two unequal elongated, cylindrical lobes or thread like structures lying on each side of the air bladder and joining together posteriorly with a common opening as a pore above the anus. The total weight of the two lobes varies between 2 to 10 g according to body weight and age. The color of the lobes brownish to light pink, opaque with very less vascularization in the tissue. By February end (Fig. 1B), the ovaries grown bigger in size with flattened lobes at the anterior end, yellowish brown in color, increased vascularization and little more transparent at the surfaces. The weight increases to 7 to 25 g. GSI ranges between 0.43 to 1.04 (average 0.78) in December as against 0.76 to 1.39 (average 1.14) in February during initiating phase (Table 1).

**Table 1:** Average Gonadosomatic index, Oocyte diameter and % immature and mature oocytes observed in the ovarian histology of rohu during resting and initiating phases

Ovarian Phases	Collection Month	GSI	Oocyte diameter (μm)	% of immature oocytes	% maturing oocytes
Resting phase	December	0.74	Type-I (21.57 to 120.51) Average: 75.77 Type-II (114.45 to 165.4) Average: 140.59	99.63 Type-I: (80%) Type-II:(20%)	Nil
Initiating phase	February	1.26	Type-I (53.58 to 133.2) Average: 89.19 Type-II (101.51 to 200.1) Average: 151.77	98.58 Type-I: (60%) Type-II:(40%)	1.42

### Histology of ovaries

*Resting phase (December):* ovary is predominantly occupied (~ 98%) by immature oocytes of both type I and II and they constitute 80 and 20% respectively, of the total immature oocyte count. Both type oocytes are having larger nucleus. However, as the size of type II cells grow larger than type I, the nucleus become prominent with a greater number of nucleoli arranged along the inner margin of the nuclear membrane. The average diameter of type I oocytes is 75.77 μm. Some type-II oocytes are also seen which are larger in size (average diameter 140.59 μm) but less in number. In this phase neither any maturing oocytes (type-III) nor any mature oocytes are observed. Immature type I oocytes are seen towards the periphery whereas large type II cells are located towards the center (Fig. 1C).

*Initiating phase (February):* Histologically, ovaries show more type-II oocytes (60%) than type I immature oocytes (40%) and both of which constitute 98.58% of the total oocyte count. The type II cells are at perinucleolar stage. The average diameter is 89.19 μm and 151.77 μm of type I and type II oocytes, respectively. Rarely, type-III maturing oocytes may be visible (1.42%) with yolk vesicles appear along the periphery in the ooplasm but no matured follicles could be observed in this phase also (Fig. 1D).

Histological analysis of ovarian tissue of PSR and IGA phases revealed the changes in response to seasonality. Primary ovarian follicles (POF), smaller in size were present during PSR dormancy (lower GSI). During IGA phase having higher GSI maturation of secondary ovarian follicles (SOF) with larger size was seen in the histological photomicrographs, revealing conspicuous seasonal differences (Fig. 1A, B, C, D). It confirmed the accuracy of tissues collected in the investigation. Similar histological attributes of ovarian development stages have also been reported in other fishes like zebrafish [9].

Like other Indian teleosts, the reproductive cycle of *L. rohita* may be divided into four stages, viz.*,* preparatory period (February–April), pre-spawning period (May–June), spawning period (July–August), and post-spawning period (September–January) and at each stage gonads show discrete changes [10]. Generally, in an adult rohu female, the ovary remains quiescent and almost inactive during last part of post spawning period also called post spawning resting (PSR). With gradual increase in day length and temperature, there is increase of gonadal activity (IGA) for preparatory period. This confirmed that these two reproductive contrasting phases, viz., PSR and IGA as most appropriate transcriptomic data points to study the changes that happens at the molecular level during onset of ovary maturation in this tropical species manifesting biological rhythm of seasonal breeding.

### Pre-processing and assembly

**Fig. 2:**
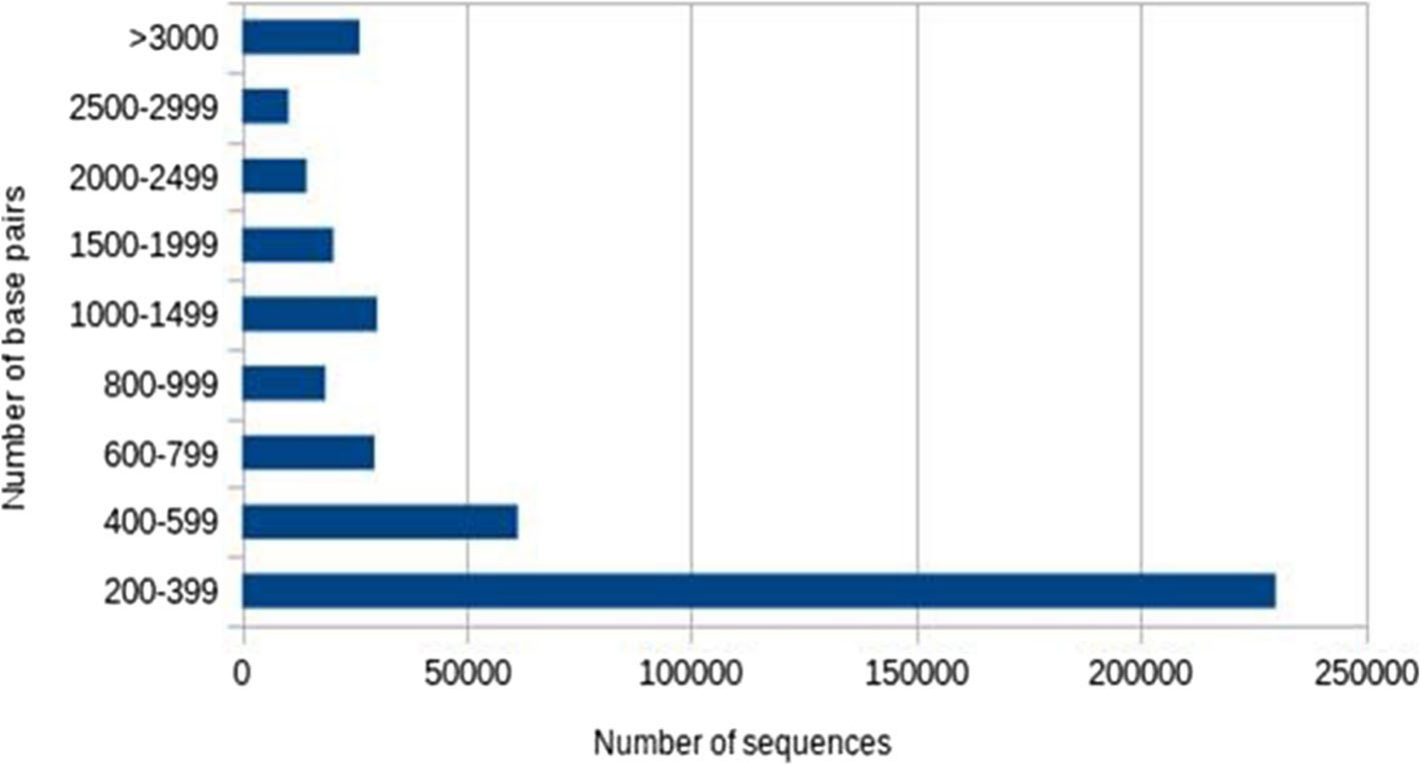
Sequence length distribution of the data

A total of ~ 150 Gb of transcriptomic data of four tissues viz., BPGL were pre-processed before assembly by removing 1,098,340 poor quality reads. The remaining 305,461,765 high quality reads were subjected to de novo assembly to obtain DEGs. A total of 440,665 transcripts were generated with *k*-mer size 25. The minimum contig length was 201 bp and maximum of 19,331 bp with average length of 867 bp. N50 was found to be 1850 bp and total GC content as 42.94% (Table 2). Figure 2 shows the sequence length distribution of the data under study. A total of 45,019 unigenes were involved in the formation of isoforms. Numbers of isoforms were found in single unigenes ranging from 2 to 30.

**Table 2:** De novo assembled statistics of Rohu transcriptome (All IGA vs. all PSR)

Total trinity transcripts	440,665
Total trinity genes	336,520
Percent GC	42.94
Contig N50	1850 bp
Minimum contig length	201 bp
Maximum contig length	19,331 bp
Median contig length	381 bp
Average contig	867.45 bp
Total assembled bases	382,252,837

### Identification of candidate genes

A total of 35,840, 21,767, 31,097 and 14,558 DEGs were identified for IGA [BR] vs. PSR [BR], IGA [PIT] vs. PSR [PIT], IGA [OVA] vs. PSR [OVA] and IGA [LIV] vs. PSR [LIV] respectively. The upregulated and downregulated DEGs from the datasets are presented in Table 3. Expression profiling and hierarchical clustering of DEGs of all tissue specific comparison were shown in the form of heatmaps. MA plots and volcano plots of differentially expressed genes were generated by edgeR tool which showed the red color dots are differentially expressed and black color dot represents the non-DEGs and all these plots were generated on the basis of log2 fold change values (Supplementary file 1).

**Table 3:** Number of upregulated and downregulated DEGs from IGA [BR] vs. PSR [BR], IGA [PIT] vs. PSR [PIT], IGA [OVA] vs. PSR [OVA] and IGA [LIV] vs. PSR [LIV]

Datasets	Upregulated	Downregulated	Total DEGs
IGA [BR] vs. PSR [BR]	14,647	21,193	35,840
IGA [PIT] vs. PSR [PIT]	10,050	11,717	21,767
IGA [OVA] vs. PSR [OVA]	17,194	13,903	31,097
IGA [LIV] vs. PSR [LIV]	6786	7772	14,558

**Fig. 3:**
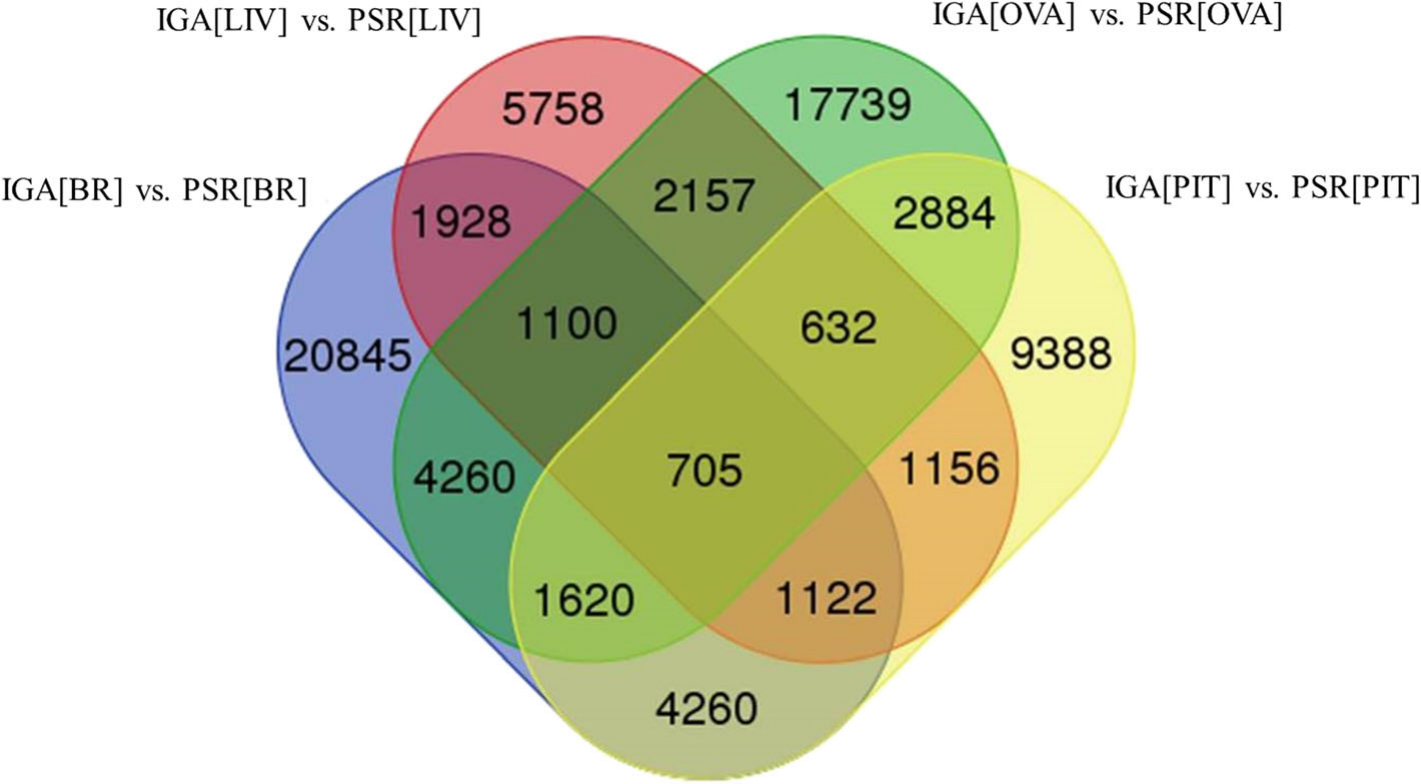
Venn diagram to identify the unique and common unigenes among all tissue specific differentially expressed genes

Venn diagram was constructed to identify the unique and common unigenes among all tissue specific candidate genes (Fig. 3). A total of 705 unigenes were found common in all datasets of tissue specific DEGs. We found 20,845, 9388, 17,739 and 5758 unigenes as unique in IGA [BR] vs. PSR [BR], IGA [PIT] vs. PSR [PIT], IGA [OVA] vs. PSR [OVA] and IGA [LIV] vs. PSR [LIV], respectively.

### Annotations and gene ontology classification of DEGs

We found 79.27, 84.38, 82.87 and 86.44% of differentially expressed genes of IGA [BR] vs. PSR [BR], IGA [PIT] vs. PSR [PIT], IGA [OVA] vs. PSR [OVA] and IGA [LIV] vs. PSR [LIV] having blast match, respectively (Supplementary file 2: Uploaded at “Supplements” tab of http://webtom.cabgrid.res.in/lrsatdb/). Table 4 described the number of sequences which showed the blast hits, mapping, interproscan and annotations statistics for dataset under study.

**Table 4:** Sequence similarity to know sequence, annotation and mapping unigenes

	IGA [BR] vs. PSR [BR]	IGA [PIT] vs. PSR [PIT]	IGA [OVA] vs. PSR [OVA]	IGA [LIV] vs. PSR [LIV]
Total Sequences	35,840	21,767	31,097	14,558
With InterProScan	35,829	21,765	31,089	14,558
With Blast Hits	28,411	18,369	25,773	12,584
With Mapping	22,625	15,306	21,196	10,708
With Annotation	17,010	11,986	15,892	8458

DEGs from all tissue specific comparison were categorized into three sub division i.e. biological process, molecular function and cellular component (Supplementary file 3). Gene ontology and KEGG (Kyoto Encyclopaedia of Genes and Genomes) analysis were performed by using Blast2Go pro software v 4.0 [11]. Tissue specific DEGs were mapped to KEGG pathways and 131, 126, 130 and 124 pathways were found in IGA [BR] vs. PSR [BR], IGA [PIT] vs. PSR [PIT], IGA [OVA] vs. PSR [OVA] and IGA [LIV] vs. PSR [LIV], respectively (Supplementary file 4).

### Prediction of transcription factors controlling seasonality genes

**Fig. 4:**
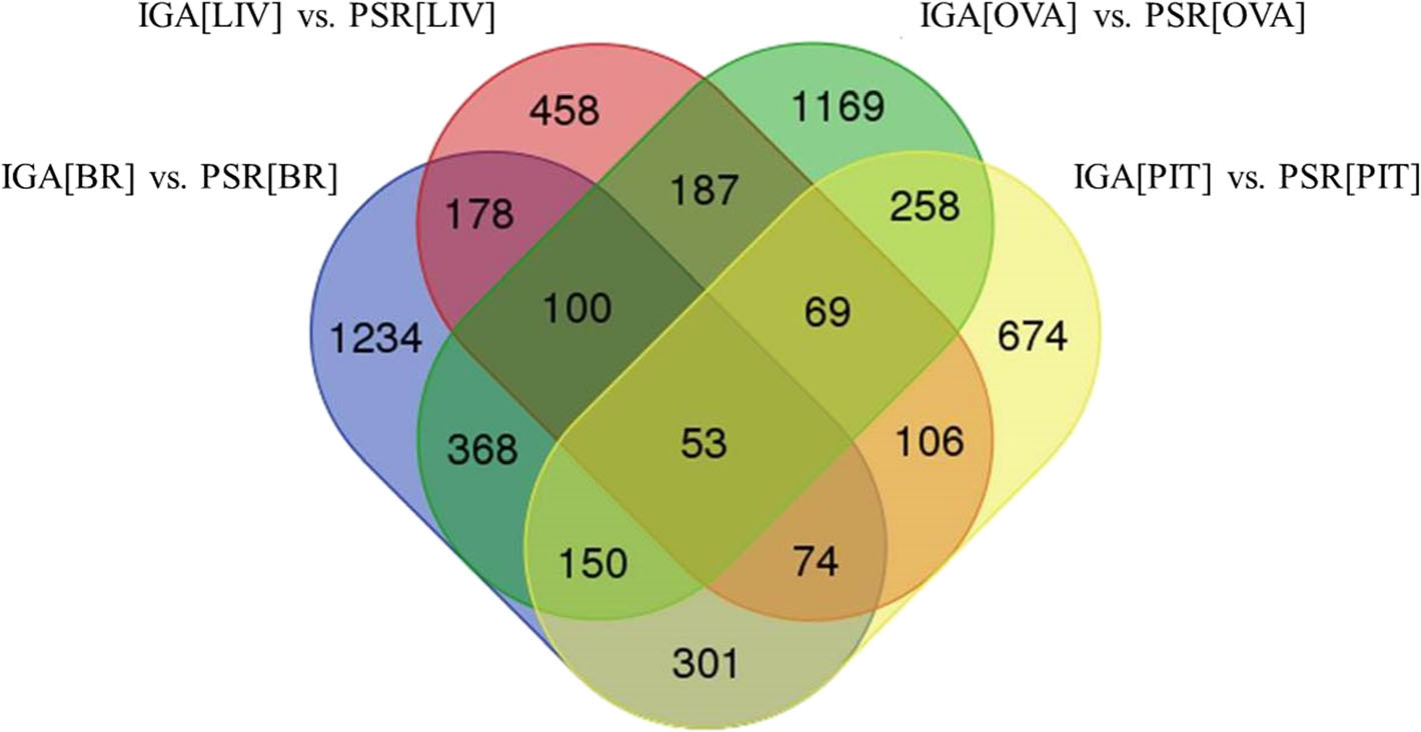
Venn diagram of transcription factors in all the tissue specific

We found 2458, 1685, 2354 and 1225 transcriptions factor (TFs) in sets IGA [BR] vs. PSR [BR], IGA [PIT] vs. PSR [PIT], IGA [OVA] vs. PSR [OVA] and IGA [LIV] vs. PSR [LIV], respectively (Supplementary file 5). Out of these transcription factors, 53 were found common in all the datasets. We also found 1234, 674, 1169 and 458 transcription factors as unique in all the tissue specific sets i.e., IGA [BR] vs. PSR [BR], IGA [PIT] vs. PSR [PIT], IGA [OVA] vs. PSR [OVA] and IGA [LIV] vs. PSR [LIV], respectively (Fig. 4). The molecular pathway controlled by these differentially expressed TFs are predicted in this study for all the four sets of data of both the phases (Supplementary file 5). Many of our reported TFs are already reported to regulate biological clock genes in flies and humans by acting as time switch genes [12]. We found some of these are well known to control BCGs and are also conserved even in this carp fish. Example of such TFs are *clock, foxl, bmal, kiss, esr, prdm, ofx, myc, srebf, bhlh* and *PAS*. These TF and/or BCGs regulates gene regulatory network (GRN) of BPGL axis manifesting circannual rhythm of breeding [13].

### Prediction of miRNA controlling seasonality genes

We identified miRNA target prediction of all tissue specific differential expressed genes with 1637 mature miRNAs of nine fish species with cut-off threshold of total score at 1000 and total energy > − 14 kcal/mol. We obtained 790, 641, 764 and 629 mature miRNAs that regulate 1444, 770, 1229 and 1044 differential expressed genes at tissue specific stages IGA [BR] vs. PSR [BR], IGA [LIV] vs. PSR [LIV], IGA [OVA] vs. PSR [OVA] and IGA [PIT] vs. PSR [PIT] (Table 5) (Supplementary Table 6). From all the tissue specific stages, we identified several miRNAs which were involved in various specific functions, like, gonadal development, oocyte and early embryo, nervous system already reported in nine other fish species (Supplementary file 7).

**Table 5:** List of mature miRNAs of nine fish species which regulate transcripts at tissue specific stages IGA [BR] vs. PSR [BR], IGA [PIT] vs. PSR [PIT], IGA [OVA] vs. PSR [OVA] and IGA [LIV] vs. PSR [LIV]

S. NO	Species	Mature miRNA	IGA [BR] vs. PSR [BR]	IGA [LIV] vs. PSR [LIV]	IGA [OVA] vs. PSR [OVA]	IGA [PIT] vs. PSR [PIT]
1	*Danio rerio*	350	163 (761)	140 (452)	152 (668)	128 (549)
2	*Fugu rubripes*	108	58 (287)	43 (176)	54,243)	41 (223)
3	*Cyprinus carpio*	146	79 (339)	64 (224)	77 (323)	62 (256)
4	*Hippoglossus hippoglossus*	37	19 (229)	14 (148)	21 (182)	14 (167)
5	*Ictalurus punctatus*	205	108 (620)	93 (358)	113 (559)	98 (147)
6	*Oryzias latipes*	146	61 (243)	47 (174)	64 (257)	50 (215)
7	*Paralichthys olivaceus*	38	14 (61)	10 (31)	16 (62)	12 (52)
8	*Salmo salar*	498	228 (1002)	185 (556)	210 (862)	181 (752)
9	*Tetraodon nigroviridis*	109	60 (293)	46 (179)	57 (279)	43 (232)

Our finding of predicted miRNA involved in reproductive seasonality fills the gap as there is no such report in rohu fish. This might be due to difficulty in isolation by cloning due to low expression, temporal expression and stability, tissue especially for smaller size RNA. Thus, computational identification of miRNAs binding site can be used for prediction and classification of the functional attributes. Instead of isolating small RNA afresh, the same larger RNA can be used to predict specific binding site to have information without any additional cost and time. Our enlisted predicted miRNAs can be used for exploration and validation of future biomarker research in this species. Such use has been reported in other fish species as marker for egg quality and embryonic development is a potential biomarker in rainbow trout [14]. These miRNAs are also reported to be involved in oocyte and early embryo [15–17] and gonadal development [18].

### Discovery of putative markers of seasonality associated genes

A total of 115,534 putative SSR markers were mined from de novo assembly of transcriptome using MISA perl script. We found 89,133, 35,931, 13,645, 3312, 73, 51 mononucleotides, dinucleotides, trinucleotides, tetranucleotides, pentanucleotides and hexanucleotides, respectively (Table 6, Supplementary file 8: Uploaded at “Supplements” tab of http://webtom.cabgrid.res.in/lrsatdb/).

**Table 6:** SSR marker of all tissues specific DEGs

	IGA [BR] vs. PSR [BR]	IGA [PIT] vs. PSR [PIT]	IGA [OVA] vs. PSR [OVA]	IGA [LIV] vs. PSR [LIV]
**Sequences examined**	35,840	21,767	31,097	14,558
**Identified SSRs**	19,633	12,899	16,461	8874
**SSR containing sequences**	13,602	8784	11,385	5979
**Sequences containing more than one SSR**	4209	2837	3520	1975
**SSRs present in compound formation**	1202	741	997	526
**Mono-**	11,173	7498	9735	5492
**Di-**	5717	3603	4465	2174
**Tri-**	2319	1563	1975	1075
**Tetra-**	406	221	269	123
**Penta-**	12	7	10	5
**Hexa-**	6	7	7	5

We obtained a total of 123,994 and 120,652 variants from IGA and PSR tissues samples, respectively against *Danio rerio* GRCz10 reference genome. A total of 99,981 variants were found common in IGA and PSR transcriptomic sets. In heterologous mapping with species *Danio rerio*, maximum numbers of variants were found on chromosome number 5. Maximum numbers of variants were 7617 and 7238 in IGA and PSR, respectively. In de novo-based mining, a total of 45,098 and 43,420 SNPs and Indels were obtained in IGA and PSR samples, respectively. Among this, 22,934 SNPs and Indels were common. In IGA samples, unigenes number c144686_g1_i1 (polyprotein) were found maximum number of times i.e., 116 and in PSR samples, c144749_g1_i1 unigene was found abundantly i.e., 77. Unigene “c144749_g1_i1” might be a novel transcript, as it did not show any specific matched with NCBI blastx (uncharacterized protein). Stringent filtering criteria was used for the detection of SNPs and Indels, i.e., minimum 15x [19] read depth and quality of 30 were used. Annotations of detected variants were performed against *Danio rerio* GRCz10 reference and identified the region where maximum number of variants were present (Supplementary file 9: Uploaded at “Supplements” tab of http://webtom.cabgrid.res.in/lrsatdb/).

### Molecular mediation of reproductive seasonality and key candidate genes

We report a total of 137 key candidate genes controlling reproductive seasonality and its major pathways operating in four tissues, viz., brain, pituitary, gonad and liver. Since major molecular pathways of reproductive seasonality is orchestrated by interplay among these four tissues, thus, we computed co-expressional network to unveil this in the form of gene regulatory networks (GRN). A GRN depicts set of genes that interact with each other to control a specific cell function pertaining to a particular trait. Such studies are important to understand development, differentiation and response to environmental stimuli. By making such network of large number of DEGs can be further narrowed down [20]. SNPs of genes involved in GRN affects phenotype/trait [21] thus are valuable for eQTL discovery. Since prediction of the behaviour of GRN are rapid and economical than wet lab experiments, thus computational methods can be a valuable research tool [22]. GRN studies has been used to identify candidate genes associated with the traits [23]. GRN is further controlled by transcription factor and microRNA [24].

The GRN models of brain, pituitary, gonad and liver tissues were represented by 21, 35, 46 and 25 genes, respectively. Some of them were predicted as hub genes having similar protein-protein interaction. Magnitude of such genes expression can be correlated with trait of interest [25]. As not much information is available on seasonality aspect of these tissues, thus limited sample size transcriptomic data was used to develop “logical model” to have a basic picture of GRN reflecting qualitative attributes of genes involved in the network rather than precise quantitative relationship [26]. Similar BPGL/BPG axis has been reported with various candidate genes controlling reproduction and its synchronization with food availability and photoperiodism in other farm fishes [27].

### Seasonality genes and their pathways in brain

Transcriptome analysis of brain revealed 21 hub genes which are further controlling network of > 80 genes to mediate signals of seasonality events in reproduction. GRN in brain controlling seasonality was constructed using top 100 co-expressed genes to depict protein-protein interactive network. Genes were selected at a cut off degree value of 167. A total of 20 hub genes were predicted having 8 up, 13 down regulated genes shown in Supplementary file 10. Since fish neuroendocrine system and brain is having very high similarity (82%) with human brain [28], thus available literature can be used to understand and explain the GRN operating in brain manifesting seasonal breeding. A set of 21 key genes which are differentially expressed in brain of rohu fish are involved in mediation of seasonality events. Major physiological and molecular events are summarised in Table 7 along with the respective key genes investigated in this tissue. Our co-expressional network reveals that brain is the key tissue right from sensing of daylength by visual stimuli, neuro modulation, neuro translation, neurotransmitter synthesis and release, integration of light and temperature regulated circadian gene expression, melanocytes and growth and learning regulation of feeding behaviour.

**Table 7:** Role of different hub genes identified in rohu fish brain

Gene	Description	Reference
ank1b and ank3b	Ankryn (ank) gene: Regulatory protein interacting with TF in cis and trans regulatory mode optimizing various physiological function via voltage-gated sodium channels, and KCNQ2/3 channels which is fast and precise as regulatory signaling	[94]
OPTN	Optinuerin (OPTN): Produced in neuroendocrine tissues which modulates beta-amyloid converting enzyme 1 involved in production of amyloid-beta	[95]
rims1 gene	Regulating synaptic membrane exocytosis 1 (rims1) gene: It is a RAS gene superfamily member which regulates neurotransmitter release by controlling synaptic vesicle exocytosis and dendrite formation by melanocytes	https://ghr.nlm.nih.gov/gene/RIMS1
PCMT	Protein-L-Isoaspartate (D-Aspartate) O-Methyltransferase (PCMT) gene plays role in monoaminergic neuromodulation, glutamatergic neurotransmission and neuronal function in development	[96]
fryA gene	Furry homolog (fryA) gene regulates cell proliferation controlling structural integrity with polarity of spindle, involved in neural development and cell adhesion. It also regulates sensory signaling involved in reproductive behavior in zebrafish and sticklebacks fish	[97]
PCLOB Gene	Piccolo (PCOLB) gene regulates neurotransmitter secretion by controlling release of synaptic vesicles in salmon and zebrafish	[98]
SMAARCC2	SWI/SNF Related, Matrix Associated, Actin Dependent Regulator Of Chromatin Subfamily C Member 2 (SMARCC2) gene is a member of the SWI/SNF family having helicase and ATPase activities regulating transcription by altering the chromatin structure	[99]
STCH gene	This is a Hsp70 family gene reported to be a promising candidate gene for sensing incubation time by ocular dominance and cortical plasticity mediating visual stimuli	[100]
AATK gene	Apoptosis associated tyrosine kinase (AATK) gene: This gene has epigenetic regulation associated with growth and differentiation. Regulates GRIN2 gene, which is one of the key genes of learning and memory in zebrafish	[101]
EIF31	Controls lipid metabolism, biosynthesis, proteolysis and gluconeogenesis in liver of zebrafish	[102]
WDFY gene	Regulates feeding behaviour and growth involved in brain-pituitary axis of zebrafish	[103]
PHF24	A well conserved gene across various phyla, involved in integration of light and temperature in the regulation of circadian gene expression	[104]

Above finding reveals that seasonality is synchronized by temperate latitudes, photoperiod, abundance of food, temperature and other environmental cues which are input to neuroendocrine output. Fish brain perceives the signal of photoperiod by melatonin pathway. Eyes are the only photoreceptive organs operating through pineal gland producing melatonin. Melatonin receptors are present in suprachiasmatic nuclei (SCN) which are the transducer of such signal acting as circadian rhythm pacemaker. Saccus vasculosus (SV) acts as a sensor of such seasonal changes in day length in fish as its removal affects the BPGL axis [29]. Photoperiod is sensed by SCN and transduced to give signal to thyroid for seasonal physiological events thus thyroid-stimulating hormone (TSH) and DIO gene also play role in seasonality [30]. There are two steps in cyclical phase of seasonality viz., PSR and IGA with slow and faster growth, respectively which is controlled by BPGL axis in rohu fish [31]. Such biological rhythm is even connected with fish auditory function for environmental cue required for reproduction and breeding especially in seasonal breeding fish [32].

### Seasonality genes and their pathways in pituitary

Transcriptome analysis of pituitary revealed 35 hub genes which are further controlling network of > 65 genes involved in mediation of reproductive seasonality. To depict the integration of molecular events of seasonality operating in pituitary, we constructed co-expressional network. This protein-protein interactive network of pituitary GRN was constructed with cut off degree of 175. In pituitary of rohu fish, we found 35 hub genes with 15 and 20 up and down regulated genes, respectively (Supplementary file 10).

Our analysis further reveals that major molecular events operating in pituitary of rohu fish. Role of rohu specific hub genes is summarized in the Table 8. Our GRN depicts how MAPK signalling pathway is controlling environmental signal mediation, gonadogenesis, retenoic acid pathway controlling gametogenesis, ovarian growth and gametogenesis, increase in cell size and proliferation, oocyte maturation and ovulation, control of BPGL axis for liver metabolism, skeletal muscle development and immunity control.

**Table 8:** Role of different hub genes identified in rohu fish pituitary gland

Gene	Description	Reference
AKAP9	A-kinase anchoring protein 9 (AKAP9) gene is involved in ovarian growth and gametogenesis by MAPK signaling pathway	[105]
ANKHD1	Ankyrin repeat and KH domain containing 1 (ANKHD1) stimulates liver growth in vitellogenesis period of zebrafish	[106]
EIF3i	Eukaryotic translation initiation factor i (EIF3i) is involved in the initiation process of protein translation. Overexpression leads to increase in cell size and proliferation. In zebrafish, it regulates development of the brain, heart, vasculature, and lateral line.	[107, 108]
Myo1b	Myosin IB (Myo1B) gene affects BPGL axis by directly affecting liver metabolism and growth in cyprinid fish, fathead minnow	[109]
FLNA	Filamin A (FLNA) regulates neuro-epilthelia cell proliferation and differentiation by regulating actin cytoskeleton and transmembrane receptor complex in cardiac and skeletal muscles development in medaka fish	[110]
dpp6B	Dipeptidyl-peptidase (dpp6B) gene is involved in retinoic acid pathway controling development of eye and germ cells in medaka fish. Retinoic acid pathway controls gametogenesis.	[111, 112]
Ik gene	Ik Gene codes for protein Red. This gene is present in zebrafish. It is involved in cell proliferation and differentiation	[113]
LAMP2	Pituitary being endocrine gland has several secretory activities. Lamp2 has been reported to control such activities of lysosomes in zebrafish	[114]
IRF2 BP	Iterferon Regulatory Factors (IRFs): In grass carp and zebrafish, this highly conserved gene has been reported to modulate innate immune response. Seasonality in breeding of fish is highly correlated with such immunity	[115]
ZGC Gene	ZGC gene is involved in oocyte maturation and ovulation in zebrafish	[116]
CMYCB	MYCB gene is involved in neuroendocrine secretion in brain and physiological regulation in liver, heart, kidney in fish (European sea bass) affecting growth and differentiation	[117]
ARHGEF	ARHGEF is also known as Rho Guanine Nucleotide Exchange Factor gene family which is present in all eukaryotes including jawless (Lampreys, Agnatha) as well as jaw (cartilaginous and boney) fishes. These are the adaptive cell signaling mediators of environmental signals in mediating seasonality	[118]
DNAJC	Also known as HSP 40 play major role in immune responses and their overexpression in liver is associated with defense mechanism	[119]
LIMA1 and LIMA1A splice variant	LIM Domain And Actin Binding 1 (LIMA1) gene is associated with nuclear receptor essential for gonado-genesis	[120]

Our above findings are duly supported by various other reports having role of pituitary in manifestation of seasonality of breeding in fish. The fish anterior pituitary secretes two gonadotrophin (GTH) hormones viz., GTH-I and GTH-II which are structurally similar to FSH and LH, respectively. GTH-II in goldfish is reported to stimulate gonadal growth, steroidogenesis, ovulation and sperm release with seasonality [33]. Pituitary regulates seasonality by variation in pituitary GnRH receptor activity and gonadal feedback mechanism in catfish and goldfish [34]. Teleost have two gonadotropins, viz., GtH beta I and II. Beta subunit synthesis is regulated by hypothalamic hormones and gonadal steroids. In pre-spawning and early maturing stage of fish beta I and in post spawning beta II predominates with seasonality. In tilapia, this activity is mediated by cAMP-PKA and PKC pathways [35] FSH and LH upregulates genes involved in steroid synthesis in fish [36]. This leads to growth of primary oocyte into secondary oocyte. FSH level remains increased in ovary along with FSH receptor gene expression in ovary from pre-spawning to post spawning transforming primary. During post spawning, i.e., vitellogenesis FSH continue to rise but during ovulation it declines with surge of LH [37]. Our finding of IGA and PSR stages, GSI and histology are in consonance of these reports.

### Seasonality genes and their pathways in ovary

Transcriptome analysis of ovary revealed 46 hub genes which are further controlling network of > 54 genes involved in mediation of reproductive seasonality. In order to depict integrated molecular pathway operating in ovary, we constructed co-expressed genes network with a cut off degree of 175. Our study reveals that in rohu fish ovary, there are 46 hub genes having 23 up and 23 down regulated genes (Supplementary file 10). Our result shows rohu fish specific molecular events which are operated by neuroendocrine mechanism synchronized with physiological events. The role of various candidate genes/ hub genes identified in rohu fish ovary are shown in Table 9.

**Table 9:** Role of different hub genes identified in rohu fish gonad

Gene	Description	Reference
ZGC gene family	ZGC gene is involved in oocyte maturation and ovulation in zebrafish	[116]
ZAR1	Zar1 is highly conserved gene also present in zebrafish, involved in growth of ovarian follicles	[121]
NASP	nuclear autoantigenic sperm protein: Expression of zona pellucida protein of primary oocytes and cell cycle regulation in zebrafish is associated with NASP gene. This gene is also reported to promote female development by repressing male-specific genes	[122]
Col12A1: Collagen Type XII Alpha 1 Chain	This is a signalling molecule associated with chondrocyte differentiation especially where collagen molecules are used in tissue remodeling during physiological growth and differentiation in zebrafish	[123]
SCML4 (Sex Comb On Midleg Like)	SCML4 is differentially expressed and its GRN study has revealed its role in development of optic, retina and olfactory epithelia with reproductive seasonality in teleost fish	[124]
ptgdsb	Prostaglandin D2 Synthase: This gene is involved in synthesis of prostaglandins which controls spawning by stimulating contraction of smooth muscles leading to ovulation in goldfish	[125]
Col 6a3	Collagen Type VI Alpha 3 Chain;There is seasonal change in collagen content in muscles of fish synchronised with swimming ability. In farm fish,swimming exercise is reported to control puberty.	[126, 127]
CDC42 gene	Cell Division Cycle 42: Fish egg yolk mainly contains vitellogenins, synthesized in liver, growing oocytes takes it from circulation by receptor-mediated endocytosis in oocyte growth	[128]
ACD	Adrenocortical dysplasia homolog (ACD), Shelterin Complex Subunit And Telomerase Recruitment: This gene controls telomeric elongation mediating mitogenic actions of estrogen in granulosa cells for ovarian growth with season	[129]

Seasonal breeding fish, *Labeo rohita* has seasonal variation in ovarian development having seven distinct stages (phases) viz., Virgin/Immature, primary growth, perinucleolar, previtellogenic (yolk vesicle), vitellogenic (post-vitellogenic), germinal vesicle break down and spawning stage [38]. In ovary, there is coordinated activities for various regulators of ovulation like proteases, protease inhibitors, progestational steroids, eicosanoids, catecholamines and vasoactive peptides [39]. Our study supports the molecular events observed in two reproductive phases of rohu with respect to ovarian development and vitellogenesis with increase in body weight.

Our revealed ovarian hub genes are majorly involved in controlling mitogenic action of estrogen in grandulosa cell of ovary, growth of ovarian follicles, oocyte maturation, chondrocyte differentiation, prostaglandin synthesis, collagen synthesis and receptor mediated endocytosis. These events are reported in study of various other fishes. Neuroendocrine mechanism regulating reproduction through hypothalamo-pituitary-gonadal (HPG) axis is evolutionarily conserved in vertebrates [40]. Neuroendocrine control of BPGL axis and its associated gene regulatory network operating in brain, pituitary, gonad and liver are well coordinated to manifest seasonality in fish breeding by sensing photoperiod using biological clock genes along with temperature and food abundance [3]. We observed LH controlled DEG (poliovirus receptor and Bloodthirsty) involved in gonad development and maturation associated genes (*ehmt2* and *racgap1*) which is also reported in rainbow trout. FSH modulates steroidogenic pathway as well as early germ cell proliferation and differentiation. We found four major pathways and their genes which are differentially expressed in BPGL axis viz., IGF pathway (insulin *gene enhancer protein,* insulin receptor, insulin-induced gene, precursor of insulin and its receptor), the TGF pathway (*amh*, *inha* and *fstl3*), the Wnt pathway (*wisp1*), and pleiotrophin (*mdka*) which are also reported in rainbow trout [3]. Insulin gene enhancer protein isl with its 3 isoforms families (Isl-1,2,3), observed in our dataset which is reported to play role in regulation of IGF pathway [41]. SV organ acts as a sensor of seasonality by sensing the day length/photoperiod by rhodopsin molecule. Thus, rhodopsin gene family must be one of the major hub in brain tissue acting as a transducer for neuroendocrine output in form of GnRH [29].

### Seasonality genes and their pathways in liver

Transcriptome analysis of liver revealed 25 hub genes which are further controlling network of > 75 genes involved in metabolism associated with reproductive seasonality. The co-expressional network was constructed with a cut off degree 165. Among 25 hub genes, 9 were up and 16 were down regulated as shown in Supplementary file 10.

Our study reveals that in liver of rohu fish, JAK/STAT signaling is major pathway controlling entire liver metabolism to manifest reproductive seasonality with synchronized events in ovarian development. This study further reveals how rohu specific genes are involved in modulation of glycolysis, gluconeogenesis, oxidative phosphorhylation, apolipo protein production, MHC response, retenoic acid, betain and GABA, immune response, cholesterol and lipid transport while meanifesting reproductive seasonality. List of key candidate genes controlling these events in rohu fish liver with their specific functions are summarized in Table 10.

**Table 10:** Role of different hub genes identified in rohu fish liver

Gene	Description	Reference
JAK1 gene	In pre- spawning, JAK/STAT signaling pathway is down regulated but in spawning phase, it is up regulated for growth and increase in innate immunity of Rainbow trout fish	[130]
PCK1	PCK1 is reported to have seasonal expression in liver of animals. In fish,this gene is associated with regulation of gluconeogenesis, oxidative phosphorylation and growth.	[131, 132]
RABGAP	RAB GTPase Activating Protein This gene is associated with seasonal apolipoprotein production in liver of teleost fish	[133]
MHC1 uja	Major Histocompatibility Complex, Class I This gene is associated with teleost immune responses	[134]
ALDH5a1	Aldehyde Dehydrogenase 5 Family Member A1 This gene is highly conserved from fish to mammal and associated with seasonal changes in retinoic acid, betaine and gamma-aminobutyric acid production in fish liver	[135]
PMT (mitogenic): Protein O-Mannosyltransferase	In zebrafish, it controls apoptotic signaling mechanisms, immune response and homeostasis in unfavourable environmental stimuli or unfavourable period for breeding, thus it plays role in synchronising the liver activity with environment and seasonality	[136]
pSAT1 is similar to GPR54	Fish GPR54–1 contains PSAT1 loci, there is evolutionary propinquity between GPR54 and kisspeptin genes, later is well known for controling seasonality	[137]
G6PCA	G6PCA is associated with JAK-STAT5 signalling pathway operating in hepatocytes having glycolysis and gluconeogenesis in tilapia fish	[138]
OSBPL2B	OSBPL2B protein is involved in regulation of cholesterol trafficking and intracellular transport of lipids in zebrafish	[139]

We also found upregulation of Wnt and Notch signaling genes in all the four tissues of BPGL axis having multifarious role including higher perceptivity of auditory system in breeding season. This is in response to estrogen induced seasonal changes [42]. Besides tissue specific GRN, there are some common set of genes which are expressed in different magnitudes in different tissue with respect to seasonality. Expression of plectin gene was found to be most abundant in liver and gonads rather than brain and pituitary. Plectin proteins are abundant (75%) in cytoskeleton of ovary, which is a major phosphoacceptor, in phosphorylation, thus important for the protein’s association with the cytoskeleton [43]. Plectin is a hemidesmosomal protein that mediates hyperproliferation of gonads and liver. It also increases the expression of estrogen receptor in fish gonads which triggers seasonal response in ovary manifesting seasonality [44]. Claudin gene was found to be upregulated in all the 4 tissues but its highest expression was found in gonads. Claudin in teleost fish is reported to be tissue specific in expression. It functions as pore forming TJ protein increasing luminal fluid accumulation and volume expansion in ovary [45] as observed in histological picture (Fig. 1).

Above findings are reflective of neuro-endochrinological and physiological mechanism which operates in all oviparous vertebrates, including fish. For such events, egg-yolk and chorionic proteins like vitellogenin and choriogenin are synthesized heterologously in the liver [46]. Our findings are in consonance of the reports in other fishes. Fish liver is reported to be involved in synthesis, degradation, transportation, and storage of lipid. These major activities are well coordinated by BPGL axis along with oocyte growth and development in tune of seasonality. The axis synchronises the food availability and ingestion behaviour along with photoperiod, temperature and gonadal maturation leading to seasonal reproduction in temperate fishes [47]. Our observation in two phases of reproductive seasonality observed in rohu is also having contrasting GSI and HSI. Similar finding is also reported in other fish where vitellogenesis associated with GSI and HSI (hepato-somatic index) due to increase in total protein, glycogen and cholesterol content of the ovary and liver and stored fat reserve, which is used for gonadal maturation [48].

### qPCR for validation of DEG

To validate the finding, qPCR analysis was performed. Relative gene expression of randomly selected 20 differentially expressed genes along with one housekeeping gene (beta actin) showed similar magnitude with the computed log fold change value except three genes (Supplementary file 11). This could be due to cross-reactivity in the designed primer [49].

### Biological clock genes in carp fish for circannual breeding

Blast hit of four sets of DEGs revealed presence of at least 32 well known biological genes in carp fish. These sets of transcripts represent carp fish specific BCG sequences which is also a direct evidence for presence of these biological clock genes in this fish. These differentially expressed BCGs in BPGL tissues are involved in manifestation of circannual rhythm of breeding. In case of European fish reproductive axis gene expression in brain is known to be modulated by photoperiod which is regulated by biological clock genes and conserved transcription factors [3].

This is the first report having transcriptomic evidence of biological clock genes in rohu mediating seasonality over BPGL axis controlling reproductive behaviour. Interestingly, all these 32 biological clock genes are well conserved from lower vertebrate like cyclostome (*Lamprey*) to higher vertebrate like mammals. In pituitary, we found differential expression of *Bmal1, Clock*, *Per1, Per2, Cry1* and *Cry2* genes sensing duration of photoperiod for synchronization in BPGL axis. This culminates in manifestation of reproductive seasonality with circannual rhythm [50].

Few already reported genes of rohu fish involved in seasonality regulation were also found in our dataset. For example *Kisspeptin* and its receptor *Kissr* [51]. We found differential expression of known sex determining and seasonality genes for example aromatase *cyp19a1a*, estrogen receptor *esr1a*, and *foxl2* in ovaries similar to Iberian cyprinid fish (*Squalius pyrenaicus)* [52] and *GnRH3*, similar to other teleost [53]*.* Similar gene family like *Kiss* and *gnrh* are reported to control reproductive axis under influence of contrasting day length as photoperiod in fish sea bass [54].

Though expression of biological clock genes are tissue specific as well as magnitude specific [55] but they are well coordinated and synchronized across various organs involved in sensing photoperiod, ambient temperature, food supplies, immunity, locomotory activity and body growth [56] enabling the fish for seasonal breeding. Isotocin-neurophysin gene was found to be upregulated in brain. Isotocin is teleost homologue of mammalian oxytocin. In case of zebra fish it has been reported that isotocin stimulates the proliferation of cells enhancing the functional activities of ionocytes. Isotocin stimulates the proliferation of cells in fish gonads [57]. Similarly, higher expression of synaptotagmin gene was also found in hypothalamus and pituitary which is involved in neurotransmitter secretion [58].

Reproductive seasonality in fish is reported to be controlled by hypothalamus/ SV organ which acts as a sensor of seasonal changes in day length [29]. Existence of this organ is already reported in *Labeo rohita* [59]. Present transcriptome based study in rohu also confirms the seasonality control by similar regulatory pathway of BPGL axis. Seasonality in tropical fishes is an adaptation to environment and its optimal growth and perpetuation of species in its specific ecological niches. Fish represents vertebrate circadian timing system operated by circadian biological clock and photoreceptor organ. This ability originates during embryogenesis and continues till reproductive age [60]. Seasonal reproductive behaviour is induced by environmental cues. Various sets of biological clock sense the environmental cues and acts as a transducer eliciting neuroendocrine mediated developmental and physiological events under coordination of BPGL axis [61].

### Evolutionary conservation of BCGs from cyclostome to eutherian mammals

**Fig. 5:**
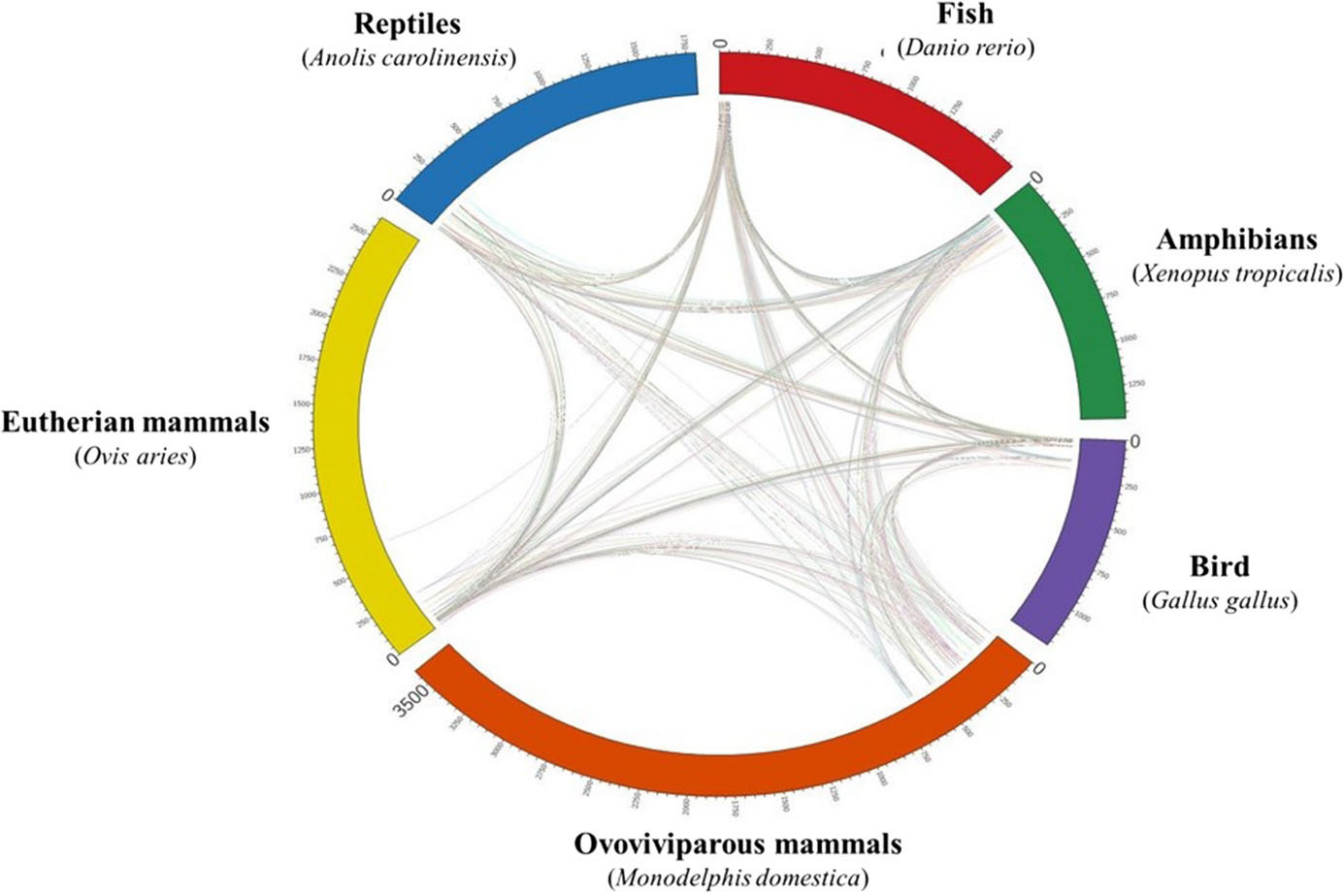
Extent of rohu biological clock gene conservation across different classes of chordates

Extent of conservation in primitive jawless fish like lamprey, cartilaginous, teleost, lung fish; amphibian, bird, reptile and three mammalian classes (monotreme, marsupials and eutherians) is summarized in Table 11. The comparative analysis of carp fish BCGs sequence with different vertebrate phyla indicates that these biological clock genes are well conserved in all. Maximum percentage similarity of all the 32 biological clock genes of rohu was found with jawless fish, lamprey. However, query coverage was maximum up to 100% with cyprinid (common carp) and zebrafish up to 100% (Supplementary file 12). Circular plot shows further qualitative depiction of extent of conservation in selected six phyla namely, amphibian, fish, birds reptiles, eutherian mammals and ovoviviparous mammals (Fig. 5).

**Table 11:** Similarity of rohu biological clock gene across different classes of chordates showing extent of conservation

Sl No.	Biological Clock Gene Name	Identity in Percentage									
		PM	RT	DR	CC	XT	GG	AC	OA	MD	OvA
1	*Bmal2*	100	76	93	94	79	76	74	75	77	76
2	*clock*	81	78	97	96	81	86	90	83	82	86
3	*cry1*	100	78	90	81	81	80	79	84	81	80
4	*cry2*	100	77	90	96	82	82	78	83	84	86
5	*foxl2*	100	83	86	85	85	90	80	85	85	87
6	*GNRH*	100	72	92	84	71	75	69	83	75	83
7	*kisspeptin*	90	91	85	86	93	93	90	85	84	89
8	*LH*	89	67	76	83	66	68	67	70	67	68
9	*PER1*	100	72	85	90	81	71	88	77	79	80
10	*Per2*	100	83	81	89	78	77	81	78	77	78
11	*Per3*	100	89	88	90	77	91	77	80	85	79
12	*esr1a*	95	83	84	91	87	86	82	84	84	85
13	*PVR*	100	77	84	90	73	70	75	74	81	68
14	*Bty*	94	69	85	87	75	88	77	69	66	70
15	*wisp1*	100	73	89	85	76	71	75	81	76	77
16	*mdka*	100	95	84	89	72	81	76	70	89	73
17	*amh*	88	91	97	87	91	82	83	88	93	91
18	*ependymin*	100	77	90	92	75	76	76	88	70	72
19	*CaMK2*	77	81	83	90	85	84	83	82	84	84
20	*ehmt1*	100	80	89	84	75	77	80	79	75	80
21	*ehmt2*	100	87	91	88	73	79	77	80	77	80
22	*racgap1*	100	73	81	85	75	79	76	81	64	77
23	*fstl3*	100	69	90	87	81	72	93	76	72	80
24	*fstl4*	93	73	75	92	66	69	83	69	90	74
25	*fstl5*	89	65	90	89	64	67	67	67	68	67
26	*prdm*	100	81	72	85	70	75	76	74	71	73
27	*Otx*	100	70	93	96	73	72	73	81	71	71
28	*Myc*	100	79	88	93	76	78	77	69	71	80
29	*srebf1*	100	75	89	87	78	78	76	82	80	75
30	*pac*	84	85	85	90	84	83	85	84	89	85
31	*picalm*	93	86	86	92	80	81	80	79	81	83
32	*aanat*	100	69	88	90	90	76	71	70	69	70

PM: *Petromyzon marinus*; RT: *Rhincodon typus* (whale shark); DR: *Danio rerio* (zebrafish); CC: *Cyprinus carpio* (common carp); XT: *Xenopus tropicalis* (tropical clawed frog); GG: *Gallus gallus* (chicken); AC: *Anolis carolinensis* (green anole); OA: *Ornithorhynchus anatinus* (platypus); MD: *Monodelphis domestica* (gray short-tailed opossum); OvA: *Ovis aries* (sheep).

These findings infer that genes and neuroendocrine pathways sensing environmental cues are well conserved during evolutionary process of species diversification into different classes of vertebrates [62]. Such biological clock genes are reported to be conserved in other fishes also like Atlantic cod fish having 18 conserved biological clock genes [63]. Thus, investigation of present transcriptomic landscape revealed that at least 32 reported biological clock genes of other diverse species are also present in rohu fish. With seasonal changes these genes are differentially expressed synchronizing environment and reproduction.

In evolution, biological clock has been found universal, which originated 2.5 billion years ago [64]. This is because of universality in response of all evolving organisms against change in day length with rotation of earth having daily and seasonal variation in environment. Environmental signal entrains the BCGs to trigger seasonal behaviour for annual biological rhythm for reproduction/migration [65]. Magnitude of biological rhythm varies from 20 min (bacterial reproduction) to daily as circadian rhythm but such rhythm may have much longer duration like tidal rhythm (14 days), lunar rhythm (28 days), circannual rhythm one year, sometime it may be of 17 years (cicada species emergence from ground) to 60 years (bamboo flowering) [66].

Circadian rhythm is well connected to lunar, tidal, circannual rhythm in hibernating mammals. It is also present in invertebrates [67]. Circannual rhythm of breeding is well known in farm fishes, birds and mammals [68]. Artificial manipulation of day length affects rhythmicity of biological clock affecting reproductive activities like ovulation, spermatogenic activity, gamete quality and sexual behaviour in fish, bird and mammals [69]. Oscillation in biological rhythm has adaptive significance to reduce competition between species by occupying differential species specific spatial niches. This is done by differential phasing of biological rhythm which is entrained by both internal and external force [8]. Thus, we observe ubiquity of circadian rhythm phylogenetically having strong adaptive significance [70]. This evolutionary comparative genomic study endorses that fish biological clock can be used as a model for research and knowledge discovery in terms of depicting molecular and cellular mechanism in response to environment in applied research like drug efficacy for jetlag etc. [71].

### Web genomic resources of evolutionary conserved BCGs and candidate genes of seasonality

Present investigation of environmentally triggered reproduction can be of much relevance in the endeavour of out of seasonal breeding leading to higher fish productivity [56]. The present genomic resource catalogues 75,554 differentially expressed transcripts, 115,534 SSRs, 65,584 SNPs from de novo assembly, 514 pathways, 5379 TFs, 187 mature miRNA regulating 1576 DEGs and 32 BCGs. It also catalogues ready to use primers. This is freely accessibility for academic purpose.

Fish transcriptome can be a powerful tool to establish relationship between genotype and phenotype using structural and functional annotation approach [72]. In rohu fish, SSRs and SNPs have been reported to be associated with important disease resistant trait against aeromoniasis bacterial disease [72]. Our transcriptome database can be used as genomic resources as it is having putative markers useful for similar association studies in future [73]. Such genomic resources having SNP markers has also been used in linkage mapping and QTL discovery in rohu fish [74].

Candidate genes involved in GRN of ovary can be used in SNP discovery and future association studies in fish selection. Liver GRN genes can be used for biomonitoring of fish breeding area with seasonality to predict magnitude of fish productivity [75]. GRN of liver having TOR signaling pathway which is associated with lipogenesis can be over-activated to utilize glucose more efficiently by genetic selection [76]. Similarly, genes expressed in liver associated with innate immune system can be used for biomonitoring of fish mortality [77].

## Conclusion

This is the first report in carp fish rohu deciphering candidate genes of circannual biological rhythm of reproduction along with BCGs. Study reveals reproductive seasonality of rohu fish with elucidation of neuroendocrine physiological events at molecular level. We present the transcriptomic landscape of BPGL axis operating through various gene regulatory network associated with tissue involved in manifestation of circannual breeding having environmental synchronization by BCGs and BPGL axis. Differentially expressed genes in BPGL tissues along with putative molecular markers (SSRs and SNPs), pathways, transcription factors, mature miRNA and BCGs are available in the form of web genomic resources (LrSATDb: http://webtom.cabgrid.res.in/lrsatdb/). This study reveals atleast 32 BCGs in this carp fish for the first time which are also highly conserved across jawless, cartilaginous, teleost fish, amphibian, reptile, birds, prototheria, marsupials and placental mammals. This is because of universal molecular mechanism of rhythmicity in response to environment and earth rotation. Biological rhythm and its amplitude has different adaptive and reproductive significance during speciation and evolutionary divergence of different taxa.

Gene regulatory network operating in BPGL tissues mediating photo-periodism sensing, neuroendocrine secretion, metabolism and yolk synthesis in liver, gonadal maturation, muscular growth with sensory and auditory perception in this fish are elucidated. Study further reveals that fish can be a good model for research on biological clock besides its immense use in the endeavour of reproductive efficiency improvement.

## Methods

### Ethics statement

As per Chapter 4, Section 15(1) of the Prevention of Cruelty to Animals (PCA) Act 1960 of Govt of India, using guidelines recommended by CPCSEA (Committee for the Purpose of Controlling and Supervising Experiments in Animals), Institute Animal Ethics Committee of ICAR-CIFA approved the experiments on 1st March 2017.

### Rearing and maintenance of the brooder in the pond

Adult male and female rohu of two-year-old (body weight 0.8 to 1.5 kg) was reared in earthen ponds of 0.05 ha in the carp breeding unit of ICAR-CIFA farm (Latitude 20°1′06″–20°11′45″N, Longitude 80°50′52″–85°51′35″E, 45 m MSL) as per standard procedure. For optimum condition, stocking density was maintained at 1500 kg/ha along with routine monitoring of water temperature, pH, dissolved oxygen, total alkalinity and total hardness at 24–30 °C, 7.5–8.5, 100–140 ppm, and 100–130 ppm, respectively. Fishes were fed with a commercial feed (CP-24%) @2% of their body weight once daily.

### Reproductive phase identification by GSI estimation with ovarian morphology and histology

Length and body weight of each euthanized individual were recorded before opening of the abdominal cavity. Fishes were humanely sacrificed as per SOP for dissection permitted by the Institute. The gonad position in the posterior half of the abdominal cavity on both side below the swim bladder was observed. Testis in male or ovary in female was identified by using the features like position of the gonadal ducts, length, appearance and color etc. Only the ovaries in females were taken out intact from the peritoneal covering, photographed and weighed. GSI was calculated using the formula: GSI = weight of the ovary X 100/ weight of body [78]. For histology, middle portion of the right and left lobes of the gonads from the sampled fish were taken after dissection. The ovary samples (1–1.5 mm thickness) were immersed in Bouin’s fixative for 24 h and 5 μm thick sections were cut using a mechanical microtome (WESWOX Optik Rotary Microtome, Ambala Cantt, India). Sections were stained using Delafield’s haematoxylin, counterstained by eosin and were processed for examination with light microscopy using routine histological procedures [79]. The diameter of the oocytes was measured by the oculometer standardized against a stage micrometer on random sampling basis.

### Tissue sample collection

The fishes were euthanized with MS-222 at 300 mg/L before dissection. Brain, pituitary, ovary and liver tissues of adult rohu female were collected during post spawning resting (PSR) phase in December and initiating gonad activity (IGA) phase in February, respectively. Tissues were collected from minimum ten fishes for each phase, quickly frozen in liquid nitrogen and stored at -80 °C, until used for RNA extraction. In order to avoid biological variability within each phase of fishes we used one ovary for RNA extraction and other one for histological and GSI confirmation.

### RNA extraction and sequencing

Total RNA was extracted using Qiagen RNeasy mini kit using manufacturer’s protocol. RNA concentration and purity were estimated using Bioanalyzer 2100 (Agilent, Santa Clara, CA, USA) and samples having RIN (RNA Integrity Number) > 7.0 were used for sequence data generation. Library preparation was performed using Illumina TruSeq RNA library protocol and sequenced on Illumina HiSeq 2000 platform (Illumina, San Diego, CA) to generate 100 nucleotide pair-end reads.

### Pre-processing, de novo assembly, abundance estimation and annotation

Data were pre-processed for quality check and low-quality reads, reads with ambiguous bases ‘N’ and adaptor sequences were trimmed using FASTQC [80] and trimmomatic [81] tool. The processed paired end reads of PSR and IGA phases were pooled together to make rohu specific de novo transcriptome assembly using Trinity [82] which was used to obtain differentially expressed genes. We preferred this transcriptome assembly rather than non-seasonal breeding zebrafish reference sequence to avoid exclusion of rohu specific novel transcripts/ genes. Further, abundance estimation was performed using RSEM (RNA-Seq by Expectation Maximization) [83] and DEGs were identified from the data set of four tissues. DEGs from comparative studies of IGA season vs. PSR season were identified in each of the BPGL tissues using edgeR package [84].

### Homology search and functional characterization of candidate genes

Standalone NCBI blast was used for sequence similarity search of all novel transcripts and differential expressed genes against the NCBI non-redundant database (nr.32). Annotation was done for the DEGs along with functional categorization, interproscan, mapping and identification of KEGG pathways using Blast2GO PRO 3.1 software [11]. Another ciprinid fish, *Danio rerio* transcription factors available in AnimalTFDB 2.0 database [85] were retrieved to predict similar transcription factors but differential expression of these genes in BPGL tissues were done using de novo transcriptome assembly of rohu.

### Discovery of putative markers of seasonality associated genes

Simple Sequence Repeats (SSRs) were detected from *L. rohita* by using MISA perl script [86]. Mononucleotides, dinucleotides, trinucleotides, tetranucleotides, pentanucleotides and hexanucleotides repeating units were identified with default parameters i.e. 10 repeating units for mono-, 6 repeating units for di-, 5 for tri-, tetra-, penta- and hexa-nucleotides.

SNPs and Indels were detected using two references i.e. *Danio rerio* reference genome data and generated de novo transcription reference assembly. The reference genome of *Danio rerio* GRCz10 was retrieved from ensemble database (http://asia.ensembl.org/Danio_rerio/Info/Index?redirect=no) as it is closely related species of *L. rohita* and both belongs to same family, *Cyprinidae*. We separately identified the SNPs and Indels from IGA and PSR tissue samples. Burrows-Wheeler Aligner (BWA) tool [87] was used to alignment and SAMtools package used for calling SNPs and Indels [88]. Annotation of SNPs and Indels were performed using SnpEff tool (http://snpeff.sourceforge.net/). Stringent filtering criteria was used for the detection of SNPs and Indels, i.e., minimum 15x [19] read depth and quality of 30 were used. Annotations of detected variants were performed against *Danio rerio* GRCz10 reference and identified the region where maximum number of SNPs were present.

### miRNA target prediction of DEGs

A total of 1637 mature miRNA sequences of nine fish species such as *Danio rerio*, *Fugu rubripes*, *Cyprinus carpio*, *Hippoglossus hippoglossus*, *Ictalurus punctatus*, *Oryzias latipes*, *Paralichthys olivaceus*, *Salmo salar* and *Tetraodon nigroviridis* were retrieved from miRBase database release 21 [89]. MiRNA target predictions of *L. rohita* from DEGs of all stages were performed by using miRanda-3.3a tool [90].

### Construction of gene regulatory network

Gene regulatory network (GRN) was constructed using up and down regulated DEGs having > 8 fold difference in expression. Visualization was done using open source tool, Cytoscape version 3.2.1 [91]. Network centrality was analysed using *Network Analyzer* plug-in. Analysis of degree, betweenness and stress were used to identify hub genes and its network. The “logical model” was constructed based on sample size [26].

### qPCR for validation of DEG

For quantitative PCR, 20 transcripts were randomly selected. Primers were designed using Primer 3 software [92]. The primer were synthesized in 10 nm scale and purified by HPLC. The total RNA were converted into cDNA using affinity script qPCR cDNA synthesis kit (Agilent Technologies, USA) as per manufacturer’s protocol. Gene expressions were measured using SYBR Green chemistry (Brilliant II SYBR Green qPCR master mix, Agilent Technologies, USA) with standard 40 cycles in Stratagene mx3005P instrument (Agilent Technologies, USA). The dissociation curve analysis was performed to ensure specificity of amplification. PCR efficiency was calibrated by 10-fold serial dilution of PCR product of each gene. Specific gene amplification was cross-checked by melt curve analysis with each primer set. Each PCR was done with technical duplicate along with negative control (without template). The mean Ct value of technical replicates were calculated. Magnitude of differential gene expression were calculated in terms of ΔΔCT fold change value as described by Pfaffl [93]. Housekeeping beta actin gene was used as reference to normalize the qPCR data. This gene was selected based on literature reporting successful use in similar targeted tissues in rohu fish [73]. Fold change values were computed by log transformation (Log2).

### Discovery of biological clock genes in carp fish

Four sets of DEG, each set obtained from each of BPGL tissue were subjected to homology search using BLAST. From literature, names of known BCGs [50, 63, 79] were used to identify carp fish specific BCG transcripts as an evidence of BCGs. These selected BCG transcripts were used for further analysis (supplementary file 12).

### Evolutionary conservation of BCGs from cyclostome to eutherian mammals

To identify biological clock genes and their extent of conservation, comparative genomics approach was used. For this study, 32 genes were selected viz. *Bmal2,clock, cry1,cry2, foxl2, GNRH, kisspeptin, LH (Luteinizing hormone), Per1, Per2, Per3, esr1a, PVR, Bty, wisp1, mdka, amh, ependymin, CaMK2, ehmt1, ehmt2, racgap1, fstl3, fstl4, fstl5, prdm, Otx, Myc, srebf1, pac, picalm* and *aanat* and homology search was done using Blastn programme of NCBI of 10 different genomes representing various classes of chordates such as jawless fish *Petromyzon marinus* (GCA_000148955.1); cartilaginous fish whale shark *Rhincodon typus* (GCA_001642345.2); teleost *Danio rerio* (GCA_000002035.4); Common carp *Cyprinus carpio* (GCA_000951615.2); Amphibia tropical clawed frog *Xenopus tropicalis* (GCA_000004195.3); Bird *Gallus gallus* (GCA_000002315.3); reptile Green anole *Anolis carolinensis* (GCA_000090745.2); egg laying mammal *Ornithorhynchus anatinus* (GCF_000002275.2), ovoviviparous mammal *Monodelphis domestica* (GCF_000002295.2), eutherian mammals *Ovis aries* (sheep) (GCA_000298735.2).

### Web genomic resources of evolutionary conserved BCGs and candidate genes of seasonality

An online relational database of rohu fish transcriptome was developed which catalogues tissue wise transcripts/contigs, putative SSRs, SNPs, Indels, transcription factors, miRNA targets representing two reproductive phases (IGA and PSR). The architecture is “three-tier architecture” viz., client-, middle- and database tier. This genomic resource is freely accessible for non-commercial use at http://webtom.cabgrid.res.in/lrsatdb/. In order to browse and query, user can go through the web pages in client tier. All the information are available in various tables corresponding to MySQL in the database tier. Server side scripting in PHP was done in the middle tier for database connectivity, query execution and fetching. In order to generate primers over selected markers, Primer3 executable was integrated at the backend.

## Supplementary Information

**Additional file 1.**

**Additional file 2.**

**Additional file 3.**

**Additional file 4.**

**Additional file 5.**

**Additional file 6.**

**Additional file 7.**

**Additional file 8.**

**Additional file 9.**

**Additional file 10.**

**Additional file 11.**

**Additional file 12.**

## Data Availability

The transcriptome dataset of the study used in this article are available in the NCBI repository with following accessions and is kept at hold till the publication. These would be made public after publication. (Bioproject: PRJNA401304; BioSamples: SAMN07602341, SAMN07602342, SAMN07602343, SAMN07602344, SAMN07602345, SAMN07602346, SAMN07602347, SAMN07602348).

## Abbreviations

BCGs: Biological clock genes
BPGL: Brain-Pituitary-Gonad-Liver
QPCR: Quantitative polymerase chain reaction
GRN: Gene regulatory networks
EST: Expressed sequence tag
SSR: Simple sequence repeats
SNP: Single nucleotide polymorphism
DEG: Differentially expressed genes
PSR: Post spawning resting
IGA: Initiation of gonadal activity
POF: Primary ovarian follicles
SOF: Secondary ovarian follicles
BR: Brain
PIT: Pituitary
LIV: Liver
OVA: Ovary
KEGG: Kyoto Encyclopedia of Genes and Genomes
TF: Transcription factors
Blast: Basic local alignment search tool
