# Supplementary material for: Revelation of candidate genes and molecular mechanism of reproductive seasonality in female rohu (Labeo rohita Ham.) by RNA sequencing
Source: BMC Genomics. 2021 Sep 22;22:685. doi: 10.1186/s12864-021-08001-6 (PMC8456608; doi:10.1186/s12864-021-08001-6)
Supplement: Supplementary file 1 — Additional file 1. [file 12864_2021_8001_MOESM1_ESM.doc]

**Revelation of candidate genes and molecular mechanism of reproductive seasonality in female rohu (Labeo rohita Ham) by RNA sequencing**

Sarika Jaiswal^1#^, Samiran Nandi^2#^*, Mir Asif Iquebal^1^, Rahul Singh Jasrotia^1^, Sunita Patra^2^, Gayatri Mishra^2^, Uday Kumar Udit^2^, Dinesh Kumar Sahu^2^, U.B. Angadi^1^, Prem Kumar Meher^2^, Padmanav Routray^2^,  Jitendra Kumar Sundaray^2^, Dhananjay Kumar Verma^2^, Paramananda Das^2^, Pallipuram Jayasankar^2^, Anil Rai^1^ and Dinesh Kumar^1*^

^1^Centre for Agricultural Bioinformatics, ICAR-Indian Agricultural Statistics Research Institute, New Delhi

^2^ICAR- Central Institute of Freshwater Aquaculture, Bhubaneswar, Odhisa

*Joint Corresponding Author

Dinesh Kumar ([dinesh.kumar@icar.gov.in](mailto:dinesh.kumar@icar.gov.in)); Samiran Nandi ([eurekhain@yahoo.co.in](mailto:eurekhain@yahoo.co.in))

^#^Authors Contributed Equally

**Supplementary file 1**: Heat map, MA and Volcano plots of 4 sets of BPGL differential expressed genes.


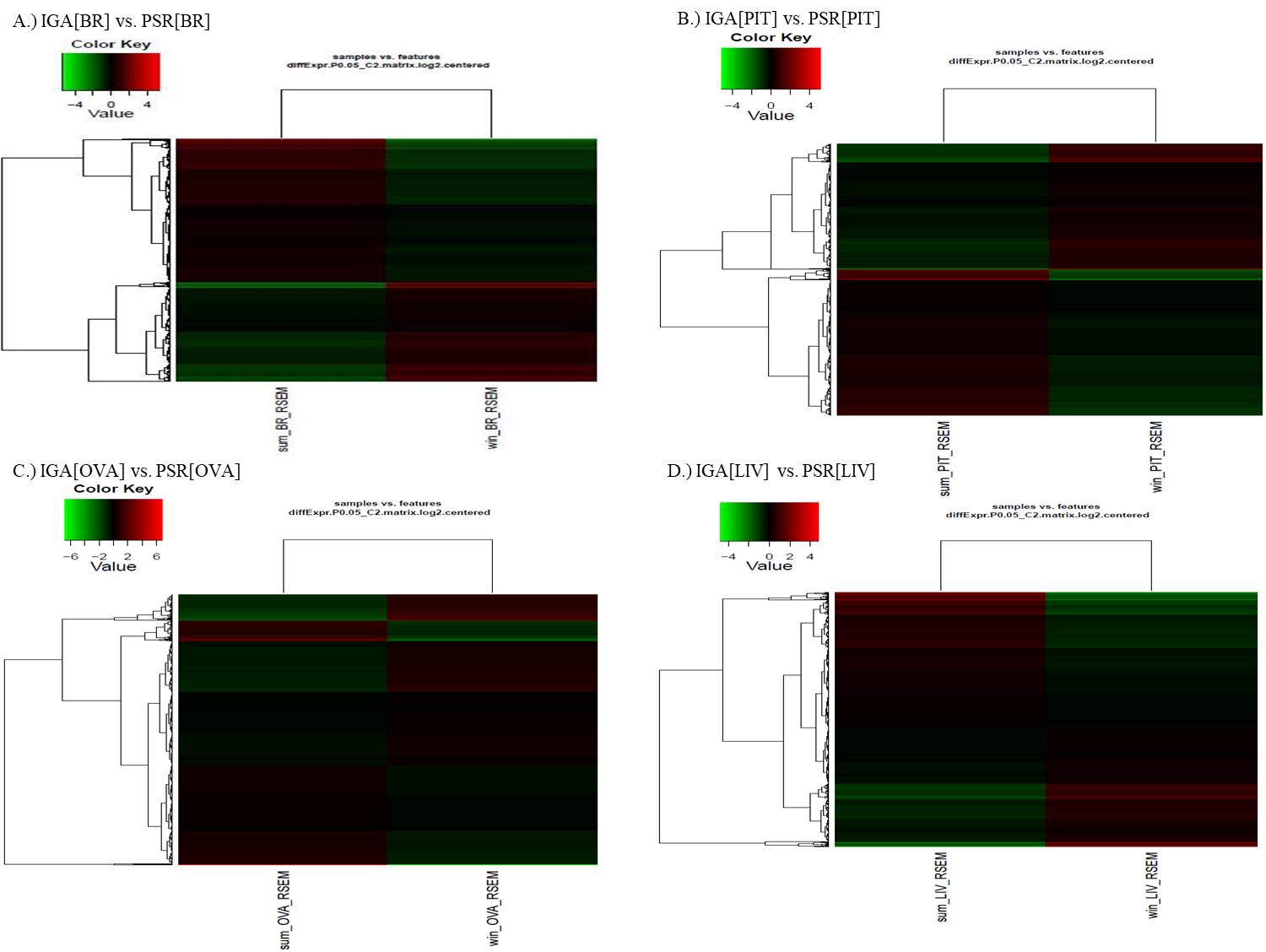


**Supplementary figure 1a**:Heatmap for the expression profiling showing hierarchical clustering of DEGs of A) IGA[BR] vs. PSR[BR]; B) IGA[PIT] vs. PSR[PIT]; C) IGA[OVA] vs. PSR[OVA]; D) IGA[LIV] vs. PSR[LIV]


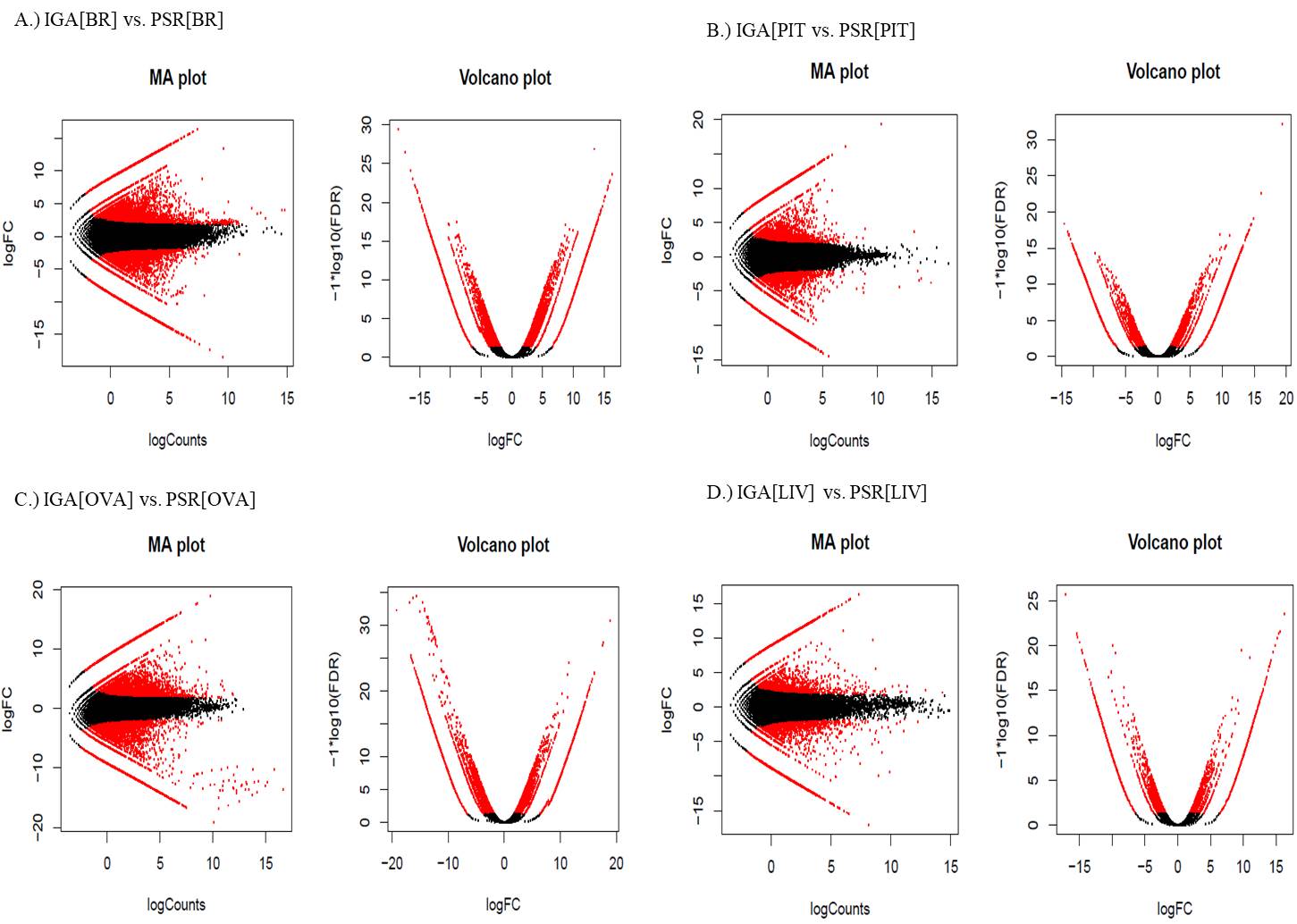


**Supplementary figure 1b:** MA and volcano plots of differentially expressed genes of A) IGA[BR] vs. PSR[BR]; B) IGA[PIT] vs. PSR[PIT]; C) IGA[OVA] vs. PSR[OVA]; D) IGA[LIV] vs. PSR[LIV] (red color dots are differentially expressed and black color dot represents the Non-DEGs).
