# Supplementary material for: Revelation of candidate genes and molecular mechanism of reproductive seasonality in female rohu (Labeo rohita Ham.) by RNA sequencing
Source: BMC Genomics. 2021 Sep 22;22:685. doi: 10.1186/s12864-021-08001-6 (PMC8456608; doi:10.1186/s12864-021-08001-6)
Supplement: Supplementary file 7 — Additional file 7. [file 12864_2021_8001_MOESM7_ESM.doc]

**Revelation of candidate genes and molecular mechanism of reproductive seasonality in female rohu (Labeo rohita Ham) by RNA sequencing**

Sarika Jaiswal^1#^, Samiran Nandi^2#^*, Mir Asif Iquebal^1^, Rahul Singh Jasrotia^1^, Sunita Patra^2^, Gayatri Mishra^2^, Uday Kumar Udit^2^, Dinesh Kumar Sahu^2^, U.B. Angadi^1^, Prem Kumar Meher^2^, Padmanav Routray^2^,  Jitendra Kumar Sundaray^2^, Dhananjay Kumar Verma^2^, Paramananda Das^2^, Pallipuram Jayasankar^2^, Anil Rai^1^ and Dinesh Kumar^1*^

^1^Centre for Agricultural Bioinformatics, ICAR-Indian Agricultural Statistics Research Institute, New Delhi

^2^ICAR- Central Institute of Freshwater Aquaculture, Bhubaneswar, Odhisa

*Joint Corresponding Author

Dinesh Kumar ([dinesh.kumar@icar.gov.in](mailto:dinesh.kumar@icar.gov.in)); Samiran Nandi ([eurekhain@yahoo.co.in](mailto:eurekhain@yahoo.co.in))

^#^Authors Contributed Equally

**Supplementary file 7**: Various species’ miRNAs characterized during different developmental stage/ tissue/process

***Danio rerio*: Zebra Fish miRNA target detect**ed in rohu

| **Tissue** | **Gonadal dev.** | **Oocyte and early embryo** | **Oocyte** | **Brain** |
| --- | --- | --- | --- | --- |
| IGA[BR] vs. PSR[BR] | _ | miR-21, miR-125a | miR-202-3p, miR-338, miR-24, miR-143, miR-148 | let-7g, let-7h, let-7i, miR-29a, miR-29b, miR-103, let-7a, let-7b, miR-21, miR-135c, miR-7b, miR-9-5p, miR-34b, miR-125a, miR-375 |
| IGA[PIT] vs. PSR[PIT] | _ | miR-125a | miR-338, miR-24, miR-143, miR-148 | let-7i, miR-29a, miR-29b, miR-103, let-7b, miR-7b, miR-9-5p, miR-34b, miR-125a, miR-375 |
| IGA[OVA] vs. PSR[OVA] | _ | miR-21, miR-125a | miR-202-3p, miR-338, miR-24, miR-143, miR-148 | let-7g, let-7h, let-7i, miR-29a, miR-29b, miR-103, let-7a, let-7b, miR-21, miR-135c, miR-7b, miR-9-5p, miR-34b, miR-125a, miR-217 |
| IGA[LIV] vs. PSR[LIV] | _ | miR-125a | miR-338, miR-24, miR-143, miR-148 | let-7i, miR-29a, miR-29b, miR-103, let-7b, miR-135c, miR-7b, miR-9-5p, miR-34b, miR-125a, miR-217, miR-375 |

***Fugu rubripes*: Fugu miRNA target detected in rohu**

|  | **Gonadal dev** | **Oocyte and early embryo** | **Oocyte** | **Brain** |
| --- | --- | --- | --- | --- |
| IGA[BR] vs. PSR[BR] | _ | miR-21, miR-125a, miR-125b | miR-221, miR-338, miR-25, miR-148, miR-202 | let-7g, let-7h, let-7i, miR-29a, miR-29b, miR-103, let-7a, let-7b, let-7d, miR-9, miR-21, miR-107, miR-125a, miR-125b, miR-128, miR-132, miR-138, miR-181b, miR-375, miR-7 |
| IGA[PIT] vs. PSR[PIT] | _ | miR-125a, miR-125b | miR-338, miR-148, miR-202 | let-7i, miR-29a, miR-29b, miR-103, let-7b, let-7d, miR-9, miR-107, miR-125a, miR-125b, miR-128, miR-132, miR-138, miR-181b, miR-375, miR-7 |
| IGA[OVA] vs. PSR[OVA] | _ | miR-21, miR-125a, miR-125b | miR-221, miR-338, miR-25, miR-148, miR-202 | let-7g, let-7h, let-7i, miR-29a, miR-29b, miR-103, let-7a, let-7b, let-7d, miR-9, miR-21, miR-107, miR-125a, miR-125b, miR-128, miR-138, miR-181b, miR-217, |
| IGA[LIV] vs. PSR[LIV] | _ | miR-125a, miR-125b | miR-338, miR-148, miR-202 | let-7i, miR-29a, miR-29b, miR-103, let-7b, let-7d, miR-9, miR-107, miR-125a, miR-125b, miR-128, miR-132, miR-138, miR-181b, miR-217, miR-375, |

***Cyprinus carpio*: Common Carp miRNA target detected in rohu**

|  | **Gonadal dev** | **Oocyte and early embryo** | **Oocyte** | **Brain** |
| --- | --- | --- | --- | --- |
| IGA[BR] vs. PSR[BR] | miR-430 | miR-21, miR-125b, miR-34 | miR-338, miR-24, miR-25, miR-143, miR-148 | let-7g, let-7i, miR-29a, miR-29b, miR-103, miR-124b, let-7a, let-7b, miR-135c, miR-7b, miR-9-3p, miR-107, miR-125b, miR-128, miR-138, miR-181a, miR-181b, miR-217, miR-375, miR-34 |
| IGA[PIT] vs. PSR[PIT] | _ | miR-125b, miR-34 | miR-338, miR-24, miR-143, miR-148 | let-7i, miR-29a, miR-29b, miR-103, miR-124b, let-7b, miR-7b, miR-9-3p, miR-107, miR-125b, miR-128, miR-138, miR-181b, miR-217, miR-375, miR-34 |
| IGA[OVA] vs. PSR[OVA] | miR-430 | miR-21, miR-125b, miR-34 | miR-338, miR-24, miR-143, miR-148 | let-7g, let-7i, miR-29a, miR-29b, miR-103, let-7a, let-7b, miR-135c, miR-7b, miR-9-3p, miR-107, miR-125b, miR-128, miR-138, miR-181a, miR-181b, miR-217, miR-34 |
| IGA[LIV] vs. PSR[LIV] | _ | miR-125b, miR-34 | miR-338, miR-24, miR-143, miR-148 | let-7i, miR-29a, miR-29b, miR-103, miR-124b, let-7b, miR-135c, miR-7b**,** miR-9-3p, miR-107, miR-125b, miR-128, miR-138, miR-181b, miR-217, miR-375, miR-34 |

***Hippoglossus hippoglossus*: Atlantic halibut miRNA target detected in rohu**

|  | **Gonadal development** | **Oocyte and early embryo** | **Oocyte** | **Brain** |
| --- | --- | --- | --- | --- |
| IGA[BR] vs. PSR[BR] | _ | miR-21 | _ | miR-21, miR-181b |
| IGA[PIT] vs. PSR[PIT] | _ | _ | _ | miR-181b |
| IGA[OVA] vs. PSR[OVA] | _ | miR-21 | _ | miR-21, miR-181b |
| IGA[LIV] vs. PSR[LIV] | _ | _ | _ | miR-181b |

***Ictalurus punctatus*: Channel catfish miRNA target detected in rohu**

|  | **Gonadal dev** | **Oocyte and early embryo** | **Oocyte** | **Brain** |
| --- | --- | --- | --- | --- |
| IGA[BR] vs. PSR[BR] | _ | miR-21, miR-30d, miR-125a, miR-125b | miR-338, miR-24, miR-25, miR-143, miR-148 | let-7g, let-7h, let-7i, miR-29b, miR-103, let-7a, let-7b, let-7c, let-7d, miR-9, miR-21, miR-135c, miR-7b, miR-34b, miR-125a, miR-125b, miR-128, miR-138, miR-181a, miR-181b, miR-375 |
| IGA[PIT] vs. PSR[PIT] | _ | miR-30d, miR-125a, miR-125b | miR-338, miR-24, miR-143, miR-148 | let-7i**,** miR-29b, miR-103, let-7b, let-7d, miR-9, miR-7b, miR-34b, miR-125a, miR-125b, miR-128, miR-138, miR-181b, miR-375 |
| IGA[OVA] vs. PSR[OVA] | _ | miR-21, miR-30d, miR-125a, miR-125b | miR-338, miR-24, miR-143, miR-148 | let-7g, let-7h, let-7i, miR-29b, miR-103, let-7a, let-7b, let-7c, let-7d, miR-9, miR-21, miR-135c, miR-7b, miR-34b, miR-125a, miR-125b, miR-128, miR-138, miR-181a, miR-181b, miR-217, |
| IGA[LIV] vs. PSR[LIV] | _ | miR-30d, miR-125a, miR-125b | miR-338, miR-24, miR-143, miR-148 | let-7i, miR-29b, miR-103, let-7b, let-7d, miR-9, miR-135c, miR-7b, miR-34b, miR-125a, miR-125b, miR-128, miR-138, miR-181b, miR-217, miR-375 |

***Oryzias latipes*: Japanese rice fish miRNA target detected in rohu**

|  | **Gonadal development** | **Oocyte and early embryo** | **Oocyte** | **Brain** |
| --- | --- | --- | --- | --- |
| IGA[BR] vs. PSR[BR] | _ | miR-125b | miR-221, miR-143 | miR-29b, miR-103, let-7a, let-7b, let-7c, miR-107, miR-125b, miR-128, miR-138, miR-7 |
| IGA[PIT] vs. PSR[PIT] | _ | miR-125b | miR-143 | miR-29b, miR-103, let-7b, miR-107, miR-125b, miR-128, miR-138, miR-92b, miR-7 |
| IGA[OVA] vs. PSR[OVA] | _ | miR-125b | miR-221, miR-143 | miR-29a, miR-29b, miR-103, let-7a, let-7b, let-7c, miR-107, miR-125b, miR-128, miR-138, miR-92b, miR-7 |
| IGA[LIV] vs. PSR[LIV] | _ | miR-125b | miR-143 | miR-29b, miR-103, let-7b, miR-107, miR-125b, miR-128, miR-138, miR-7 |

***Paralichthys olivaceus*: Japanese flounder miRNA target detected in rohu**

No miRNAs found in the species

***Salmo salar*: Atlantic salmon miRNA target detected in rohu**

|  | **Gonadal development** | **Oocyte and early embryo** | **Oocyte** | **Brain** |
| --- | --- | --- | --- | --- |
| IGA[BR] vs. PSR[BR] | _ | _ | miR-202-5p | _ |
| IGA[PIT] vs. PSR[PIT] | _ | _ | miR-202-5p | _ |
| IGA[OVA] vs. PSR[OVA] | _ | _ | miR-202-5p | _ |
| IGA[LIV] vs. PSR[LIV] | _ | _ | miR-202-5p | _ |

***Tetraodon nigroviridis*: Pufferfish miRNA target detected in rohu**

|  | **Gonadal development** | **Oocyte and early embryo** | **Oocyte** | **Brain** |
| --- | --- | --- | --- | --- |
| IGA[BR] vs. PSR[BR] | _ | miR-21, miR-125a, miR-125b | miR-221, miR-338, miR-24, miR-25, miR-25, miR-148, miR-202 | let-7g, let-7h, let-7i, miR-29a, miR-29b, miR-103, let-7a, let-7b, let-7d, miR-9, miR-21, miR-107, miR-125a, miR-125b, miR-132, miR-138, miR-181b, miR-375 |
| IGA[PIT] vs. PSR[PIT] | _ | miR-125a, miR-125b | miR-338, miR-24, miR-148 | let-7i, miR-29a, miR-29b, miR-103, let-7b, let-7d, miR-9, miR-107, miR-125a, miR-125b**,** miR-132, miR-138, miR-181b, miR-375 |
| IGA[OVA] vs. PSR[OVA] | _ | miR-21, miR-125a, miR-125b | miR-221, miR-338, miR-24, miR-148, miR-202 | let-7g, let-7h, let-7i, miR-29a, miR-29b**,** miR-103, let-7a**,** let-7b, let-7d, miR-9, miR-21, miR-107, miR-125a, miR-125b**,** miR-138, miR-181b, miR-217 |
| IGA[LIV] vs. PSR[LIV] | _ | miR-125a, miR-125b | miR-338, miR-24, miR-148, miR-202 | let-7i, miR-29a, miR-29b, miR-103, let-7b, let-7d, miR-9, miR-107, miR-125a, miR-125b, miR-132, miR-138, miR-181b, miR-217, miR-375 |
